# Neutrophil‐Driven Cascade‐Targeted Nanocarriers Restore Mitochondrial Homeostasis to Ameliorate Renal Ischemia–Reperfusion Injury

**DOI:** 10.1002/advs.202520940

**Published:** 2026-03-30

**Authors:** Hangbin Ma, Yang Li, Shen Lin, Feifan Chu, Gaozhan Ren, Yinhui Mao, Mingzhi Wu, Yuning Ma, Qiwei Ji, Zujie Chen, Jinzhong Ji, Mingxin Sun, Yongpeng Xu, Xiaoli Sun, Longguang Tang, Hao Zhou

**Affiliations:** ^1^ Department of Urology Center for Reproductive Medicine The Fourth Affiliated Hospital of School of Medicine, and International School of Medicine International Institutes of Medicine Zhejiang University Yiwu China; ^2^ Department of Pharmacy Center for Regenerative and Aging Medicine The Fourth Affiliated Hospital of School of Medicine, and International School of Medicine International Institutes of Medicine Zhejiang University Yiwu China

**Keywords:** drug delivery systems, kidney injury, mitochondrial homeostasis, neutrophil‐KIM1 dual‐targeting, NMN

## Abstract

Renal ischemia‐reperfusion injury (IRI) is a major cause of acute kidney injury (AKI), with high mortality and a significant risk of progression to chronic kidney disease (CKD). To address the lack of targeted therapies, we developed NKN‐LNP, a cascade‐targeting drug delivery system that enables spatiotemporally controlled delivery to injured tubules. This system first targets neutrophils via a surface polypeptide, hijacking their inflammatory migration to traverse the glomerular barrier and reach the injury site. Microenvironmental matrix metalloproteinase 2/9 (MMP‐2/9) then triggers nanoparticle release, exposing a second peptide that selectively binds to upregulated kidney injury molecule‐1 (KIM1) on tubular cells. Loaded with the NAD^+^ precursor β‐nicotinamide mononucleotide (NMN), the accumulated NKN‐LNP potently activates the NAD^+^‐SIRT3 signaling axis, restoring mitochondrial function and ameliorating renal damage in AKI mice. Importantly, this targeted strategy also exerts potent antifibrotic effects, thereby mitigating the AKI‐to‐CKD transition. This neutrophil‐mediated dual‐targeting platform offers a promising nanotherapeutic strategy for precise treatment of renal IRI and its chronic progression.

## Graphical abstract

A neutrophil‐mediated dual‐targeting liposome (NKN‐LNP) is developed to treat renal ischemia–reperfusion injury. The system enables spatiotemporally controlled delivery of NMN to injured tubules via MMP‐2/9 activation and KIM1 recognition, restoring mitochondrial function through NAD^+^‐SIRT3 signaling and preventing the transition from acute kidney injury to chronic kidney disease.

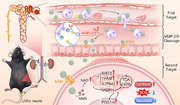

## 1 Introduction

Acute kidney injury (AKI) is a complex and multifactorial clinical syndrome characterized by an abrupt loss of renal function and associated with high rates of morbidity and mortality [1, 2, 3]. Commonly induced by ischemic or nephrotoxic insults, AKI not only leads to acute renal dysfunction but also acts as a key driver of chronic kidney disease (CKD) progression [4, 5]. Despite its considerable clinical burden, current management remains primarily supportive, reflecting the lack of effective disease‐modifying therapies [6]. The lack of sensitive early diagnostic biomarkers and mechanistically targeted interventions continues to limit therapeutic efficacy and contributes to suboptimal outcomes [7, 8]. These unmet needs underscore the urgency of developing drug delivery systems capable of targeting injured renal tubules at early stages of disease, enabling timely and localized therapeutic intervention.

Immune cells are increasingly being explored as carriers for targeted drug delivery, offering a precise means of delivering therapeutic agents to specific sites in various diseases [9, 10, 11, 12, 13]. Immune cell–derived or immune cell–hijacking nanotherapeutic systems have recently emerged as effective strategies for treating ischemia–reperfusion injury by enabling targeted delivery to injured tissues [14, 15]. Neutrophil infiltration is a defining feature of AKI induced by IRI in both clinical settings and experimental animal models [16, 17]. After renal ischemia‐reperfusion injury, neutrophils are swiftly recruited from the peripheral circulation to the site of damage, particularly in the early stages [18, 19, 20]. Exploiting the rapid recruitment of neutrophils to injured kidneys offers a novel strategy for targeted drug delivery [21]. Moreover, neutrophil‐mediated phagocytosis can be harnessed to load therapeutic molecules or nanoparticles in vitro, or to directly transport drugs in vivo by hitchhiking on neutrophils, thereby autonomously guiding targeted delivery to inflamed regions, such as those affected by renal ischemia‐reperfusion injury [22, 23, 24]. Despite the promise of these approaches, they face several challenges, including significant limitations in navigation accuracy and spatiotemporal control of drug release, as well as the inability of nanoparticles to detach from neutrophils in vivo, which impedes their ability to efficiently reach and target the injured renal tubules.

Recent studies have underscored the critical role of nicotinamide adenine dinucleotide (NAD^+^) depletion in the progression of AKI, contributing to disruptions in mitochondrial function, energy metabolism, and activation of inflammatory pathways in renal cells [25, 26, 27]. Supplementation with NAD^+^ precursors, such as nicotinamide mononucleotide (NMN), has shown promise in restoring NAD^+^ levels and delaying the transition from AKI to CKD [28, 29, 30]. However, the nonspecific absorption of NMN by peripheral tissues necessitates high doses and frequent administration, increasing the risk of adverse effects [31, 32]. To address this limitation, nanomaterial‐based delivery systems have been extensively investigated to boost renal NAD^+^ levels and mitigate AKI and subsequent AKI‐to‐CKD transition. For instance, the Fe_3_O_4_ nanoparticle‐loaded NMN system developed by Zhang et al. (2021) and the GA‐NAD NPs developed by Ying et al. have demonstrated enhanced NAD^+^ delivery and alleviated kidney damage [33, 34]. Nevertheless, these systems still encounter major challenges, particularly the demand for precise early‐stage targeting of injured renal tubules.

**FIGURE 1:**
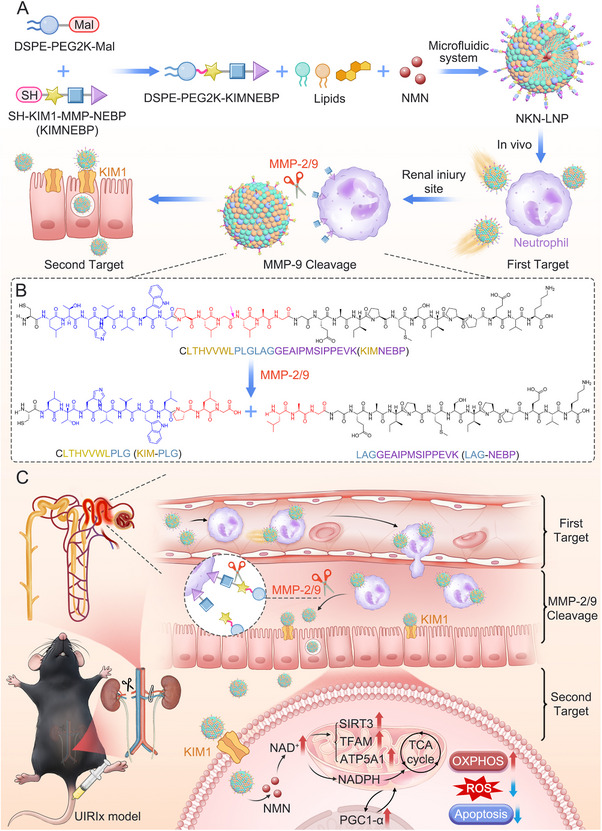
Schematic of the NKN‐LNP structure and its therapeutic mechanism for ischemia‐reperfusion injury‐induced AKI. (A) Fabrication strategy for neutrophil‐ and KIM1‐targeting peptide‐functionalized liposomal nanocarriers encapsulating NMN. Selective peptide exposure occurs only under high MMP‐2/9 expression, where NKN‐LNP expose the KIM1‐targeting moiety, enabling precise targeting of injured renal tubular epithelial cells. (B) Structural schematic of the multifunctional targeting peptide KIMNEBP. KIMNEBP is a fusion peptide composed of three sequential functional domains: the KIM1‐targeting motif (CLTHVVWL), the MMP‐2/9–cleavable linker (PLGLAG) essential for cascade activation, and the neutrophil‐binding motif NEBP (GEAIPMSIPPEVK). Together, these segments form the integrated sequence CLTHVVWLPLGLAGGEAIPMSIPPEVK, enabling dual targeting of neutrophils and KIM1‐expressing injured renal tubules. (C) Proposed mechanism of action: Following intravenous administration, NKN‐LNP initially localize to the injury microenvironment via neutrophil‐targeting moieties. Elevated MMP‐2/9 activity at the inflammatory site triggers peptide cleavage, facilitating detachment from neutrophils. Subsequently, KIM1‐targeting peptides enable selective binding to injured tubular epithelial cells, where internalized NMN restores NAD^+^ levels, activates SIRT3/PGC1‐α pathway, and attenuates mitochondrial dysfunction. This cascade dual‐targeting strategy combats apoptosis and ROS, and ultimately mitigates tubular damage in ischemia‐reperfusion acute kidney injury.

In this study, we introduce a dual‐cascade responsive nanoparticle system specifically designed to target activated neutrophils and KIM1‐expressing injured renal tubules, thereby enhancing kidney‐specific drug delivery for AKI. Elevated expression of metalloproteinase MMP‐2/9 at ischemic injury sites in AKI provides a promising trigger for controlled drug release [35, 36, 37]. To exploit this, we engineered a polypeptide (CLTHVVWLPLGLAGGEAIPMSIPPEVK(KIMNEBP)) capable of recognizing both neutrophils and KIM1‐expressing injured tubules, incorporating an MMP‐2/9‐sensitive cleavage mechanism. The polymer DSPE‐PEG2K‐Maleimide was then employed to conjugate with KIMNEBP, and then mixed with DSPC (1,2‐distearoyl‐sn‐glycero‐3‐phosphocholine), cholesterol, DOTAP (1,2‐dioleoyl‐3‐trimethylammonium‐propane (chloride)), DSPE‐mPEG2K, and NMN to form Liposome Nanoparticles (NKN‐LNPs) (Figure 1A). These nanoparticles utilize activated neutrophils for targeted delivery, allowing them to navigate to injured renal tissue and initiate the first phase of therapy. Upon reaching the injury site, MMP‐2/9 upregulation triggers the dissociation of nanoparticles from neutrophils, exposing KIM1‐targeting peptides and enabling NMN transport into damaged tubules for the second phase of release (Figure 1A,B). This cascade enhances NAD^+^ levels, preserves mitochondrial homeostasis, reduces oxidative stress (ROS) accumulation, inhibits apoptosis, and ultimately mitigates renal tubular injury in AKI, hindering its progression to CKD (Figure 1C).

## 2 Results and Discussion

### 2.1 Dynamics of Neutrophil Recruitment and KIM1 Expression in Ischemia‐Reperfusion Kidney Injury: Implications for Targeted Therapy

**FIGURE 2:**
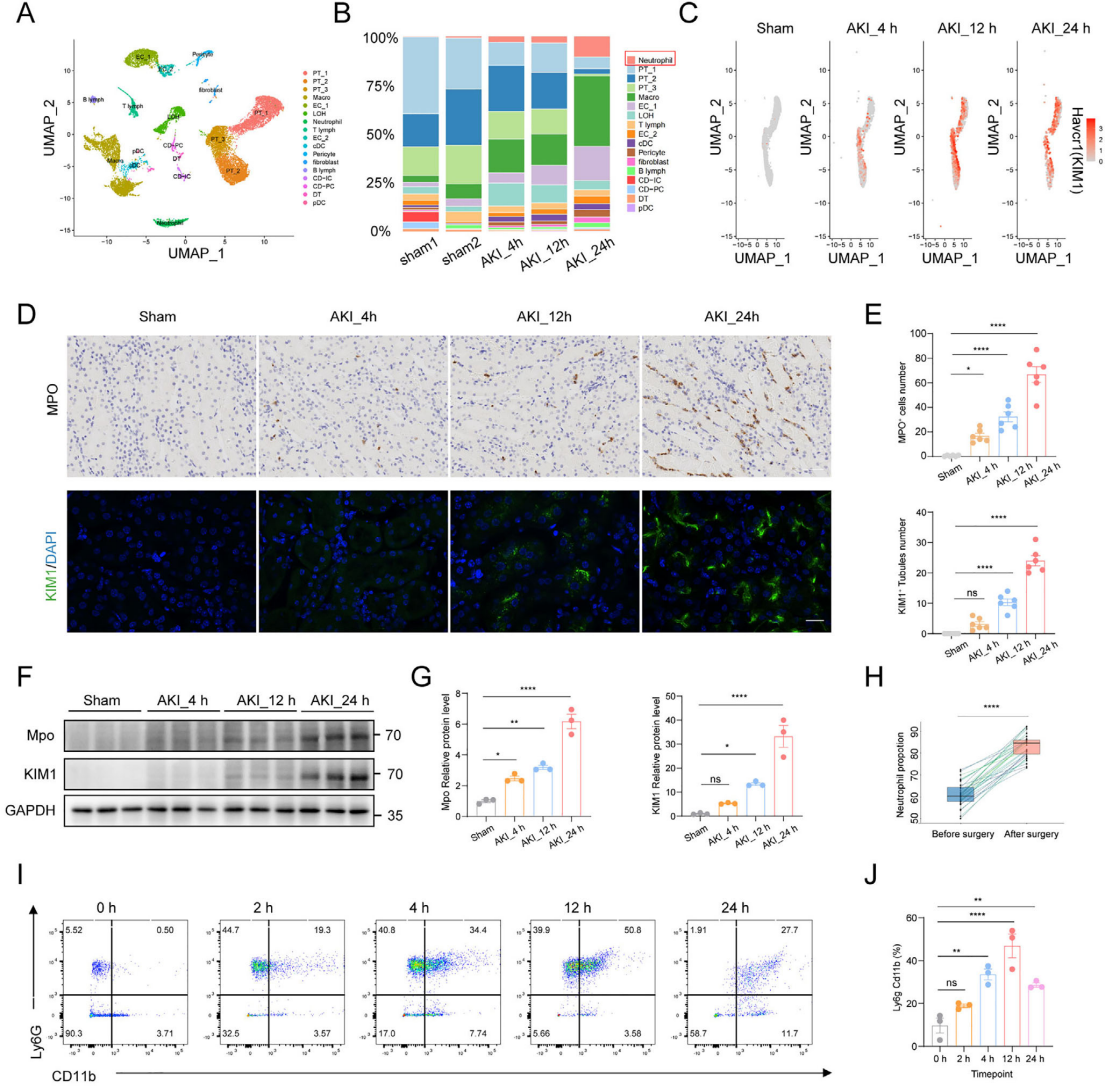
Temporal Dynamics of Neutrophil Infiltration and Tubular KIM1 Expression in Ischemic Kidney Injury. (A) UMAP visualization of scRNA‐seq data (GSE274819) from mouse kidneys after ischemia–reperfusion injury, with major cell types annotated. (B) Proportion of neutrophils across time points (0, 4, 12, and 24 h) in scRNA‐seq data. (C) Temporal expression of kidney injury molecule‐1 (KIM1) in proximal tubular cells derived from scRNA‐seq datasets at the indicated time points post‐injury. (D) Representative immunohistochemistry (IHC) staining for MPO (Scale bars: 50 µm) and immunofluorescence (IF) for KIM1 in kidney sections. Nuclei were counterstained with DAPI (blue). (Scale bars: 20 µm). (E) Quantification of MPO^+^ cells and KIM1^+^ tubule from (D). Data are presented as mean ± SEM; n = 6 mice per group; ^*^ *p* < 0.05, ^****^ *p* < 0.0001, ns no significant difference versus 0h; one‐way ANOVA with Tukey's post hoc test. (F) Western blot analysis of MPO and KIM1 protein levels in kidney lysates. Tubulin was used as a loading control. (G) Protein quantification of (F). Data are presented as mean ± SEM; n = 3; ^*^ *p* < 0.05, ^**^ *p* < 0.01, ^****^ *p* < 0.0001 versus. 0 h; one‐way ANOVA with Tukey's post hoc test. (H) Proportion (%) of neutrophils in the peripheral blood of renal partial nephrectomy patients before and after surgery (n = 29). ^****^ *p* < 0.0001; paired t‐tests. Flow cytometry analysis (I) and statistical analysis (J) of neutrophils in peripheral blood at different time points in the UIRIx model. Data are presented as mean ± SEM (n = 3); one‐way ANOVA with Tukey's post hoc test, ns, no significant difference. ^**^ *p* < 0.01 and ^****^ *p* < 0.0001.

Analysis of single‐cell RNA sequencing (scRNA‐seq) data from ischemia‐reperfusion (I/R)‐induced kidney injury tissue (GSE274819) reveals distinct cellular clusters and changes in cell proportions (Figure 2A, Figure S1A) [38]. Neutrophil subpopulations demonstrate rapid recruitment characteristics following I/R‐induced kidney injury, with their cellular proportions showing a step‐wise increase at 4, 12, and 24 h post‐injury (Figure 2B, Figure S1B). Simultaneously, the classic injury marker KIM1 exhibits time‐dependent upregulation in renal tubular epithelial cells (Figure 2C, Figure S1C). Immunohistochemical and immunofluorescence analyses reveal that MPO^+^ neutrophil infiltration in the renal interstitium increases in parallel with the number of KIM1^+^ renal tubules (Figure 2D,E). Western blot results also demonstrate that protein levels of both KIM1 and the neutrophil‐specific marker MPO (myeloperoxidase) in kidney tissues gradually increase over time (Figure 2F,G). Additionally, clinical complete blood count results from patients who underwent partial nephrectomy (with transient ischemia) show significantly elevated neutrophil proportions post‐operatively (Figure 2H). Subsequently, we established an I/R‐induced AKI model: contralateral nephrectomy followed by unilateral ischemia‐reperfusion (UIRIx). Peripheral blood flow cytometry analysis indicates a significant increase in CD45^+^/Ly6G^+^/CD11b^+^ neutrophils in the UIRIx model, with levels peaking at 12 h, further supporting the critical role of neutrophils in the pathogenesis of I/R‐induced AKI (Figure 2I,J). Based on these findings, we propose developing a novel drug delivery system for AKI intervention employing a cascade dual‐targeting approach to neutrophils and KIM1, with the aim of providing more specific and timely intervention strategies for early clinical treatment.

### 2.2 Design and Characterization of MMP‐2/9‐Responsive Cascade‐Targeting Nanoparticles for Acute Kidney Injury

**FIGURE 3:**
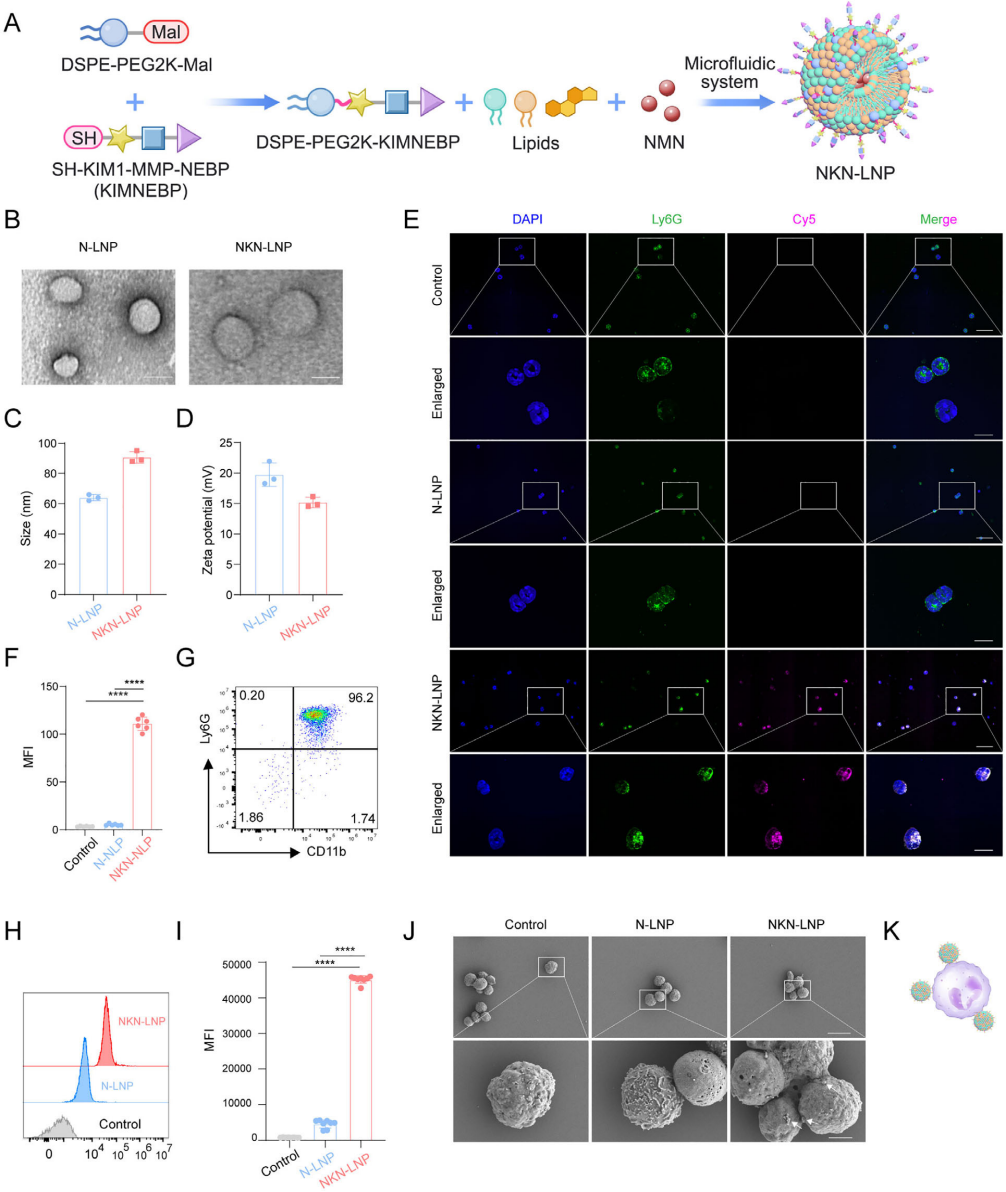
Preparation and characterization of nanoparticle properties. (A) Schematic illustration of the fabrication process of Liposome Modified with Neutrophil‐ and KIM1‐Targeting peptides. (B) TEM images of N‐LNP and NKN‐LNP (scale bar: 50 nm). (C) The liposome size was measured by dynamic light scattering (DLS) of N‐LNP and NKN‐LNP. (D) The liposome Zeta potential measured by DLS of N‐LNP and NKN‐LNP. (E) Representative confocal images of Cy5‐labeled nanoparticles (magenta) targeting Ly6G+ neutrophils (Ly6G, green); nuclei are counterstained with DAPI (blue). Individual channels and merged views are shown for Control, N‐LNP, and NKN‐LNP. White boxes mark regions of interest; lower panels show enlarged insets. Scale bars: 40 µm (low magnification); 10 µm (enlarged magnification). (F) Quantified mean fluorescence intensity (MFI) from confocal images in (E). Data are shown as mean ± SEM (n = 6). One‐way ANOVA with Tukey's post hoc test. ^****^ *p* < 0.0001. (G) Flow cytometric assessment of bone marrow neutrophil purity. Cells were gated on Ly6G^+^CD11b^+^ population. (H) Superior neutrophil binding of Cy5‐NKN‐LNP (red) compared to N‐LNP (blue). (I) Quantified MFI from (H). Data are presented as mean ± SEM; n = 6; One‐way ANOVA with Tukey's post hoc test. ^****^ *p* < 0.001. (J) Representative SEM images of neutrophils incubated with the indicated nanoparticles (Control, N LNP, NKN LNP). White boxes denote regions of interest; lower panels show magnified views of the boxed areas. Scale bars: 10 µm (low magnification), 2 µm (enlarged magnification). (K) Schematic diagram of nanoparticle binding to neutrophils.

The synthesis pathway of NKN‐LNP is depicted in Figure 3A. The MMP‐2/9‐responsive KIMNEBP peptide (sequence CLTHVVWLPLGLAGGEAIPMSIPPEVK) was synthesized through the strategic conjugation of three functional peptide motifs: the neutrophil‐targeting NEBP peptide (sequence GEAIPMSIPPEVK), the MMP‐2/9‐cleavable sequence (PLGLAG), and the KIM1‐targeting peptide (sequence CLTHVVWL). The identity and high purity of the KIMNEBP peptide were confirmed by mass spectrometry and HPLC analysis, respectively (Figure S2 A,B). Subsequently, DSPE‐PEG‐KIMNEBP was constructed via site‐specific maleimide‐thiol coupling between the KIMNEBP polypeptide and DSPE‐PEG2K‐maleimide. The resulting polymer was then integrated with NMN and lipid components to assemble a liposome (NKN‐LNP) (Figure 3A). For comparison, we also synthesized a non‐target liposome (N‐LNP) for NMN encapsulation. The particle sizes of NKN‐LNP and N‐LNP, as characterized by transmission electron microscopy (TEM) and dynamic light scattering (DLS), were uniformly distributed in the range of 60–100 nm (Figure 3B,C). The polydispersity index (PDI) of non‐targeted N‐LNP was 0.152 ± 0.018, while that of targeted NKN‐LNP was 0.153 ± 0.010 (mean ± SD, n = 3), indicating a narrow size distribution and good nanoparticle uniformity. In addition, the zeta potentials of the two nanoparticles were approximately +15 and +20 mV, respectively, attributed to the incorporation of the cationic lipid DOTAP (Figure 3D). Moreover, the liposome exhibited a high NMN loading content of 94.4 µg mg^−^^1^ and an encapsulation efficiency of 94.4%, indicating efficient drug incorporation (Figure S3A–D). To assess the neutrophil‐targeting capability of the nanomaterials, neutrophils were isolated from mouse bone marrow and subsequently activated with fMLP (10 nm), a widely utilized N‐formylmethionine peptide. The activated neutrophils were then co‐incubated with the nanomaterials for six hours. Laser scanning confocal microscopy was employed to evaluate the specificity of nanoparticle targeting toward neutrophils. The results indicated that Cy5‐labeled NKN‐LNP was fluorescently co‐localized with Ly6G‐labeled neutrophils, while Cy5‐labeled N‐LNP did not show any co‐localization with neutrophils, demonstrating that NKN‐LNP is capable of targeting neutrophils successfully (Figure 3E,F). Flow cytometry analysis confirmed the isolation of high‐purity neutrophils from mouse bone marrow (Figure 3G). Furthermore, NKN‐LNP exhibited significantly enhanced binding affinity to activated neutrophils compared to *N*‐LNP (Figure 3H,I). Scanning electron microscopy (SEM) observations also confirmed that NKN‐LNP effectively bound to neutrophils, providing additional morphological evidence of their targeting capability (Figure 3J,K). To evaluate the stability of surface‐conjugated peptides under physiological conditions, NKN‐LNP were incubated in serum‐containing culture medium for 12 h, during which no appreciable changes in particle size or zeta potential were observed (Figure S4A,B). In addition, the nanoparticles maintained stable size distributions after incubation in serum‐containing medium for up to 7 days, indicating good long‐term colloidal stability (Figure S4C,D). This finding substantiates that NKN‐LNP selectively targets neutrophils with high specificity.

**FIGURE 4:**
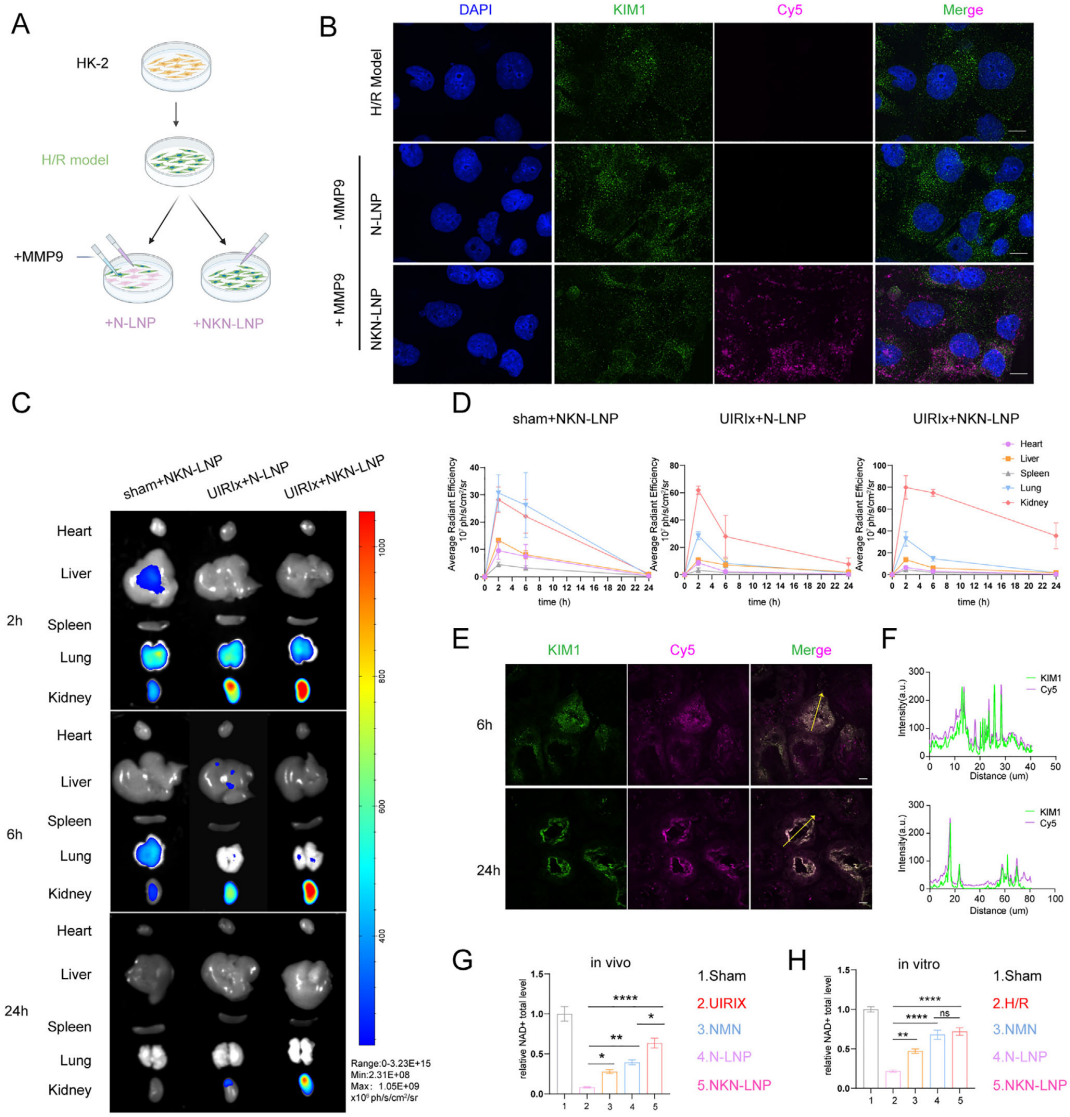
Renal‐targeted delivery of NKN‐LNP in AKI mice. (A) Schematic diagram of Hypoxia/Reoxygenation Model Construction and Treatment Application. (B) Evaluation of nanoparticles targeting KIM1‐positive HK‐2 cells upon MMP‐2/9 cleavage (Scale bars: 10 µm). (C) Ex vivo fluorescence images showing the biodistribution of nanoparticles in the major organs of sham mice and AKI mice at different times (2, 6, and 24 h). (D) Fluorescence Intensity Quantification Across Tissues at Different Time Points (2, 6, and 24 h). (E) Co‐localization of NKN‐LNP (Cy5, red) and KIM1 (green) in injured renal tubules. Nuclei: DAPI (blue). Scale bars: 10 µm. (F) Fluorescence intensity profile along the line shown in (E). The x‐axis represents the distance, and the *y*‐axis shows the relative fluorescence intensity. (G) Quantification of total NAD^+^ levels in the UIRIx model under various treatments, relative to the control group. Data are presented as mean ± SEM (n = 6); one‐way ANOVA with Tukey's post hoc test ^*^ *p* < 0.05, ^**^ *p* < 0.01, ^****^ *p*<0.0001. (H) Total NAD^+^ levels in differently treated HK‐2 cells after H/R, normalized to the control group. Data are presented as mean ± SEM (n = 5); one‐way ANOVA with Tukey's post hoc test, ^**^ *p* < 0.01, ^***^ *p* < 0.001.

NKN‐LNP exhibit MMP‐2/9‐responsiveness, enabling exposure of the KIM1‐targeting ligand specifically under conditions of elevated MMP‐2/9 expression. To recapitulate the KIM1 upregulation characteristic of AKI, an in vitro hypoxia/reoxygenation (H/R) model was established using HK‐2 cells. Cells were subsequently treated with or without MMP‐2/9 (5 µm) and incubated with the nanoparticles for 6 h (Figure 4A). Targeting efficiency was evaluated via confocal laser scanning microscopy. Upon MMP‐2/9 activation, Cy5‐labeled NKN‐LNP were effectively internalized by HK‐2 cells, confirming MMP‐2/9‐dependent exposure of the KIM1‐binding motif and enhanced cellular uptake. The results demonstrated that Cy5‐labeled NKN‐LNP exhibited fluorescence and co‐localized with KIM1 following MMP‐2/9 enzyme activation. Notably, significantly enhanced Cy5 fluorescence intensity in MMP‐2/9‐treated cells confirmed successful KIM1 targeting by the nanoparticles (Figure 4B). Importantly, after incubation with mouse plasma for 4 h, NKN‐LNP retained efficient neutrophil targeting as well as MMP‐2/9‐responsive KIM1 targeting, whereas non‐targeted N‐LNP showed negligible association, demonstrating that plasma exposure did not compromise peptide stability or targeting functionality (Figure S5A,B).

### 2.3 Cascade‐Targeting Nanoparticles Demonstrate Preferential Accumulation in Injured Kidney Tubules

We assessed the in vivo targeting capability of nanoparticles by administering Cy5‐labeled NKN‐LNP or *N*‐LNP to UIRIx model mice and sham‐operated controls, with parallel administration of Cy5‐labeled N‐LNP. Ex vivo organ imaging at defined time points (2, 6, and 24 h) revealed injury‐dependent biodistribution patterns. In sham‐operated mice, nanoparticles predominantly accumulated in the lungs and kidneys. In contrast, UIRIx mice exhibited significant nanoparticle enrichment specifically within pathological kidneys. Notably, compared with *N*‐LNP, the dual‐targeted NKN‐LNP demonstrated substantially enhanced renal retention at all monitored time points (2, 6, 24 h post‐injection), confirming superior targeting efficacy toward injured renal tissue. Furthermore, time‐dependent attenuation of fluorescence signals in major organs of sham‐operated mice indicated favorable biodegradation kinetics of the nanoparticles (Figure 4C,D).

To investigate subcellular localization, we performed high‐resolution confocal microscopy on renal cryosections. Fluorescence analysis demonstrated precise co‐localization between Cy5‐labeled nanoparticles and KIM1 (Figure 4E). The fluorescence intensity profile along a representative tubule revealed a clear spatial overlap between the signal from Cy5‐labeled NKN‐LNPs and the KIM1‐positive injured tubules. The coincident peaks in the profile demonstrated successful accumulation of NKN‐LNPs at the sites of tubular injury (Figure 4F). In contrast, representative immunofluorescence images of healthy kidneys showed no detectable KIM1 expression and minimal Cy5 fluorescence at 6 and 24 h post‐injection, with a lower Cy5 signal observed at 24 h compared with 6 h (Figure S7A). The selective accumulation in damaged tubules provides compelling evidence that our KIMNEBP peptide‐functionalized liposome effectively traverses the glomerular filtration barrier and preferentially targets injured epithelium through the proposed cascade‐targeting mechanism. Given NMN's role as a key NAD+ biosynthetic precursor, we quantified NAD^+^ dynamics across treatment groups in both hypoxia‐reoxygenation (H/R) cellular models and UIRIx mouse models. In the UIRIx group, both nanoparticles enhanced renal NAD^+^ levels, with NKN‐LNP demonstrating superior NAD^+^ augmentation attributable to its enhanced in vivo targeting specificity (Figure 4G). Consistently, in H/R‐treated cells, N‐LNP and NKN‐LNP significantly elevated intracellular NAD^+^ concentrations (Figure 4H).

### 2.4 NKN‐LNP Attenuate Ischemia‐Reperfusion‐Induced Acute Kidney Injury

**FIGURE 5:**
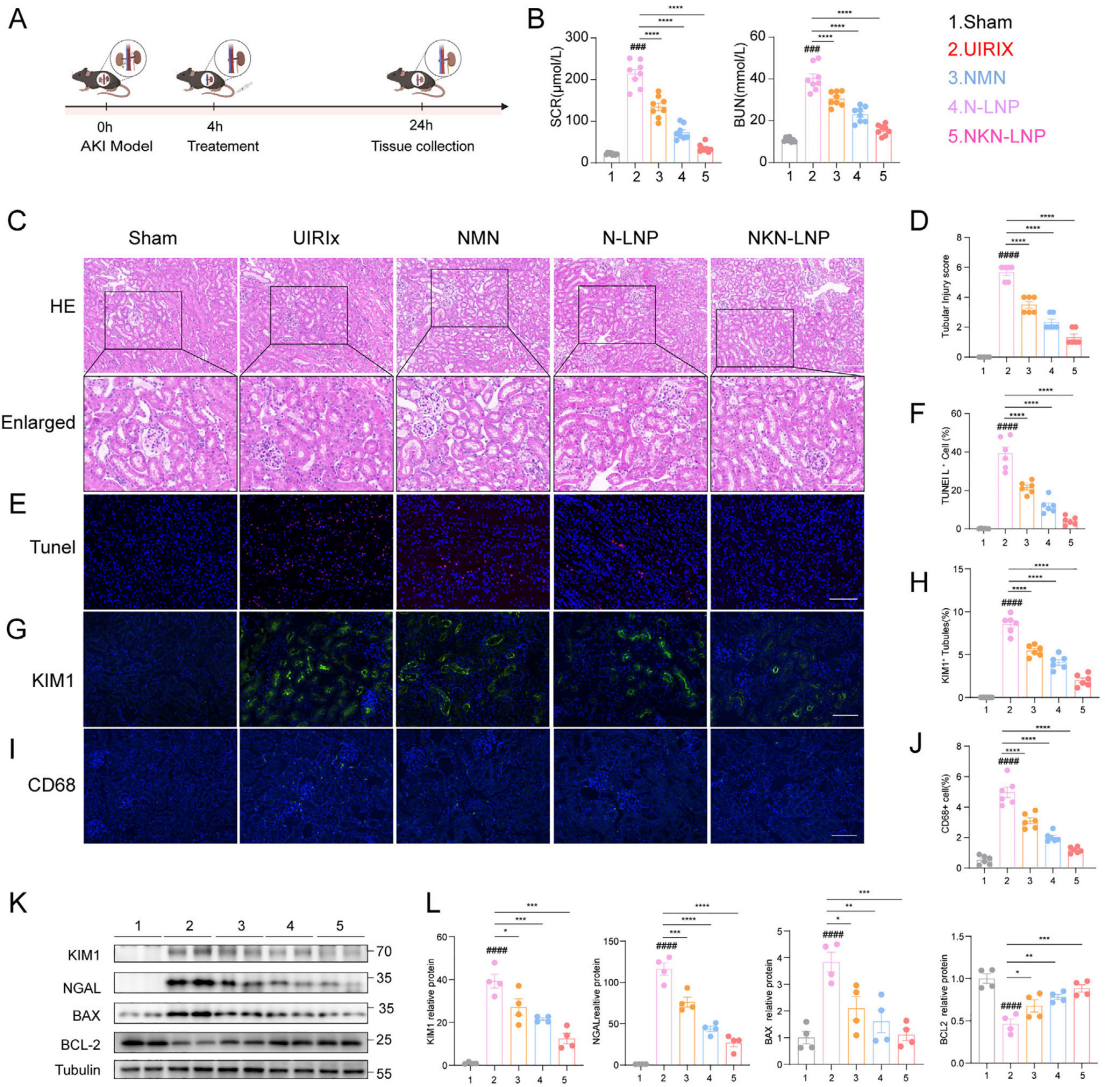
NKN‐LNP alleviated AKI and suppressed apoptosis in I/R‐induced renal injury. (A) Schematic diagram of AKI Modeling and Treatment. (B) Serum creatinine (SCr) and blood urea nitrogen (BUN) levels in AKI mice at 24 h post‐treatment (n = 8). (C) Hematoxylin and eosin (HE) staining of kidney tissues from each group, and (D) corresponding tubular injury scores (n = 6). (E) TUNEL staining, (G) KIM1 immunofluorescence, and (I) CD68 immunofluorescence in kidney tissues across groups. (F), (H), (J) Quantification of TUNEL^+^ cells, KIM1^+^ tubules, and CD68^+^ cells based on (E), (G), and (I), respectively (n = 6). (K) Western blot analysis of KIM1, NGAL, BAX, and BCL‐2 protein levels in kidney lysates from different groups, with tubulin as a loading control. (L) Densitometric quantification of KIM1, NGAL, BAX, and BCL‐2 expression levels (n = 4). In (B), (D), (F), (H), (J), and (L), data are presented as mean ± SEM. Statistical significance was determined by one‐way ANOVA followed by Tukey's post hoc test. ^*^ *p* < 0.05, ^**^ *p* < 0.01, ^***^ *p* < 0.001, ^****^ *p* < 0.0001 versus UIRIx. ^####^ *p* < 0.0001 UIRIx versus Sham.

In this study, we investigated the renoprotective effects of NKN‐LNP in the UIRIx model. After establishing the UIRIx model, different drug treatments were applied 4 h post‐injury, and therapeutic efficacy was assessed 24 h later (Figure 5A). We conducted a comprehensive series of experiments to evaluate renal protection across various treatment groups. The experimental design included: (1) sham‐operated group, (2) UIRIx model group with saline treatment, (3) NMN treatment group, (4) N‐LNP treatment group, and (5) NKN‐LNP treatment group.

Compared to the sham group, serum creatinine (SCr) and blood urea nitrogen (BUN) levels were significantly elevated in the model group. All therapeutic interventions reduced SCr and BUN levels, with the NKN‐LNP treatment group showing the most pronounced decrease (Figure 5B). These findings underscore the significant efficacy of NKN‐LNP in ameliorating renal dysfunction during AKI progression.

Histopathological evaluation revealed marked tubular injury, with luminal cast formation and cellular debris accumulation in saline‐treated UIRIx model mice (Figure 5C). In contrast, mice treated with NKN‐LNP exhibited significantly attenuated tubular damage, characterized by preserved epithelial architecture and minimal necrosis (Figure 5D). Concurrently, TUNEL staining revealed renal cell apoptotic activity, showing that NKN‐LNP‐treated mice exhibited the lowest number of apoptotic cells (Figure 5E,F).

Immunofluorescence analysis further demonstrated substantial downregulation of KIM1 across all treatment groups, with the NKN‐LNP group showing the most pronounced reduction in KIM1 expression (Figure 5G,H). Additionally, NKN‐LNP treatment significantly reduced CD68^+^ inflammatory cell infiltration in the treatment groups. Furthermore, NKN‐LNP administration markedly attenuated CD68^+^ macrophage infiltration, suggesting anti‐inflammatory renoprotection (Figure 5I,J).

Consistent with histopathological improvements, immunoblotting revealed that the protein levels of KIM1 and neutrophil gelatinase‐associated lipocalin (NGAL) were significantly reduced in NKN‐LNP‐treated mice compared with other treatment groups and the injury model group. Furthermore, the NKN‐LNP treatment group exhibited the highest levels of the anti‐apoptotic protein BCL‐2 and the lowest levels of the pro‐apoptotic protein BAX (Figure 5K,L). These findings highlight the protective efficacy of NKN‐LNP in ameliorating renal dysfunction and preserving kidney integrity following ischemia‐reperfusion injury.

### 2.5 Transcriptomic Analysis Reveals Molecular Mechanisms Underlying NKN‐LNP Treatment in Renal Ischemia‐Reperfusion Injury

**FIGURE 6:**
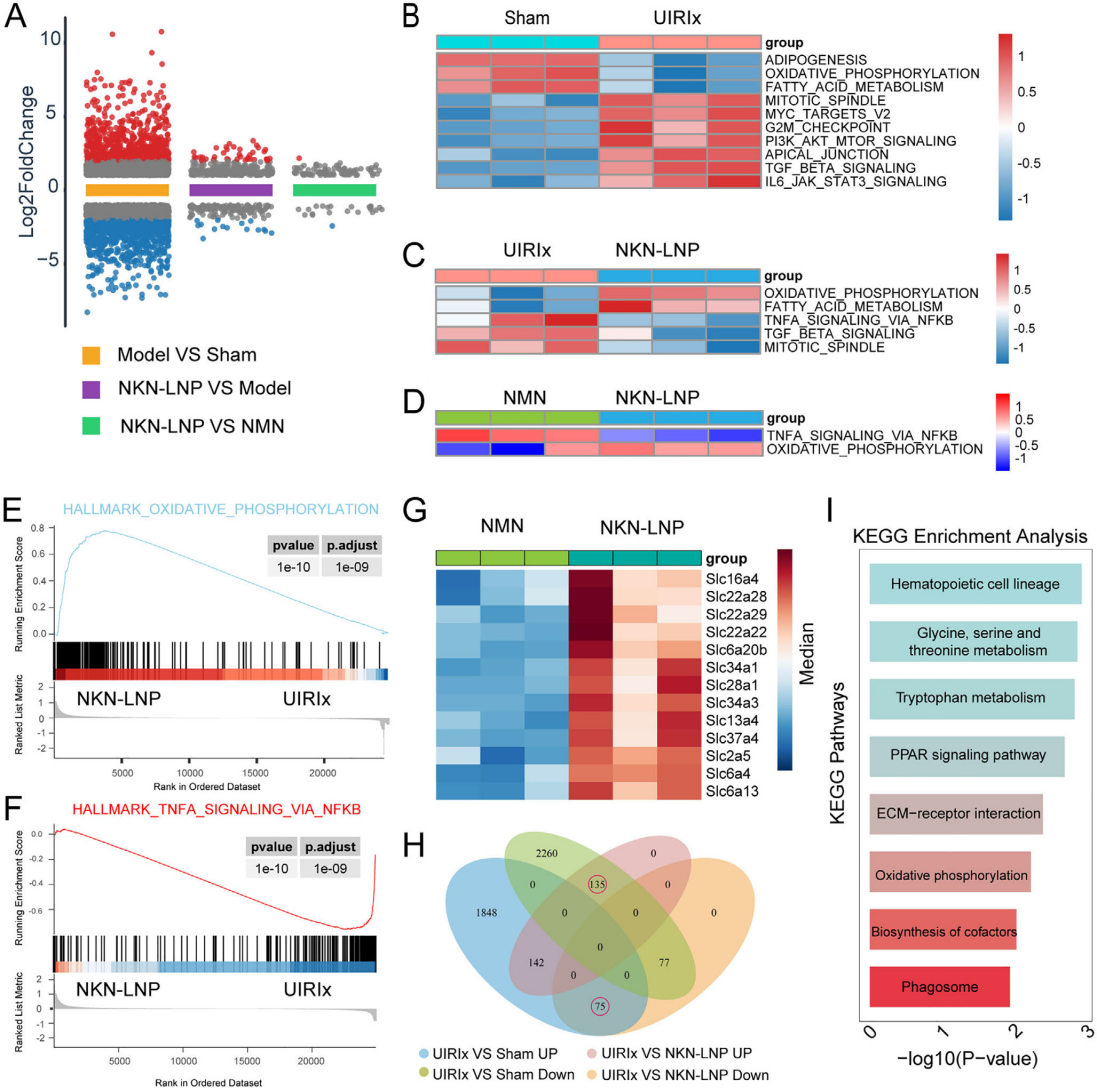
Transcriptomic analysis identifies molecular mechanisms of NKN‐LNP efficacy in renal IRI. (A) Volcano plots illustrating differentially expressed genes (DEGs) across the three groups (thresholds: |log_2_ fold change| > 1.5, false discovery rate (FDR) < 0.05). (B–D) Gene Set Variation Analysis (GSVA) comparing the Sham versus UIRIx groups, UIRIx versus NKN‐LNP groups, and NMN versus NKN‐LNP groups. Pathway differences were assessed using the limma package, with pathways showing a *p* value < 0.01 depicted in the heatmap. (E,F) Gene Set Enrichment Analysis (GSEA) of the oxidative phosphorylation (OXPHOS) in the NKN‐LNP group and TNFA SIGNALING VIA NFKB signaling pathways in the UIRIx group, with significance defined as nominal *p* < 0.05, and *p*. adjust (FDR) < 0.05. (G) Heatmap of differentially expressed genes related to the SLC (solute carrier) family between the NMN and NKN‐LNP groups. (H) Venn diagram showing the overlap of DEGs between the UIRIx model versus Sham comparison and the NKN‐LNP versus UIRIx model comparison. (I) KEGG pathway enrichment analysis of DEGs, including both upregulated and downregulated genes.

To elucidate the multiscale molecular mechanisms underlying the renoprotective effects of NKN‐LNP in attenuating ischemia‐reperfusion injury, a comprehensive RNA‐sequencing analysis was performed on kidney tissues harvested from four experimental groups (Sham, UIRIx model, NMN, and NKN‐LNP), with triplicate biological samples per group. Principal component analysis (PCA) showed that the groups were effectively separated, indicating distinct transcriptomic profiles among them (Figure S8A). Differentially expressed genes (DEGs) were rigorously identified using dual threshold criteria (log_2_ fold change > 1.5; adjusted p‐value < 0.05), revealing distinct transcriptional signatures among treatment groups (Figure 6A). GSVA analysis was then conducted between the Sham and model groups, model and NKN‐LNP groups, and NMN and NKN‐LNP groups, followed by differential pathway analysis using the DESeq2 package. Notably, we observed that the OXIDATIVE PHOSPHORYLATION pathway was upregulated, while TNFA_SIGNALING_VIA_NFKB pathway was downregulated in all three groups (Figure 6B–D). Additionally, GO and GSEA enrichment analyses were performed to further investigate the pathways altered between the UIRIx model and NKN‐LNP groups (Figure S8B,C). GSEA enrichment analysis revealed the downregulation of TNFA_SIGNALING_VIA_NFKB pathway and significant upregulation of OXIDATIVE PHOSPHORYLATION pathway in the NKN‐LNP group, suggesting potential molecular mechanisms underlying the renoprotective effects of our engineered nanoparticles (Figure 6E,F). Western blot analysis further revealed that NKN‐LNP induced the most pronounced suppression of TNFα expression among all treatments (Figure S9A,B). Differential gene analysis between NKN‐LNP and conventional NMN treatment groups identified significant upregulation of solute carrier (SLC) family transporters in the nanoparticle‐treated group (Figure 6G). Specifically, SLC16a4, encoding an organic acid transporter crucial for lactate transport, was markedly upregulated [39]. Additionally, SLC22a28, a homolog within the SLC22 transporter family, exhibited significant upregulation [40, 41]. This transporter is essential for the trafficking of critical antioxidants, including uric acid, ergothioneine, carnosine, and carnitine [42, 43]. These findings suggest that NKN‐LNP may partially confer renoprotection against acute kidney injury (AKI) through enhanced expression of these key transport proteins, although further investigation into the underlying mechanisms is warranted. To identify key functional genes modulated during kidney injury and subsequent NKN‐LNP treatment, we analyzed overlapping differentially expressed genes (DEGs) across conditions. This approach revealed 135 genes exhibiting an up‐down (UD) expression pattern—upregulated following UIRIx but downregulated after NKN‐LNP treatment—and 75 genes displaying a down‐up (DU) pattern—downregulated after UIRIx but upregulated following NKN‐LNP treatment (Figure 6H). The restoration of physiological expression levels in these gene sets suggests they represent critical targets through which NKN‐LNP exert their protective effects. KEGG enrichment analysis of these DEGs identified several relevant pathways, including oxidative phosphorylation pathways (Figure 6I). These findings were further corroborated by gene set variation analysis (GSVA) and gene set enrichment analysis (GSEA) results, collectively indicating that NKN‐LNP mitigates kidney injury primarily through enhancement of oxidative phosphorylation pathways, representing a promising therapeutic strategy for renal protection.

### 2.6 NKN‐LNP Restore Mitochondrial Homeostasis through SIRT3/PGC1α Axis in IRI‐Induced AKI

**FIGURE 7:**
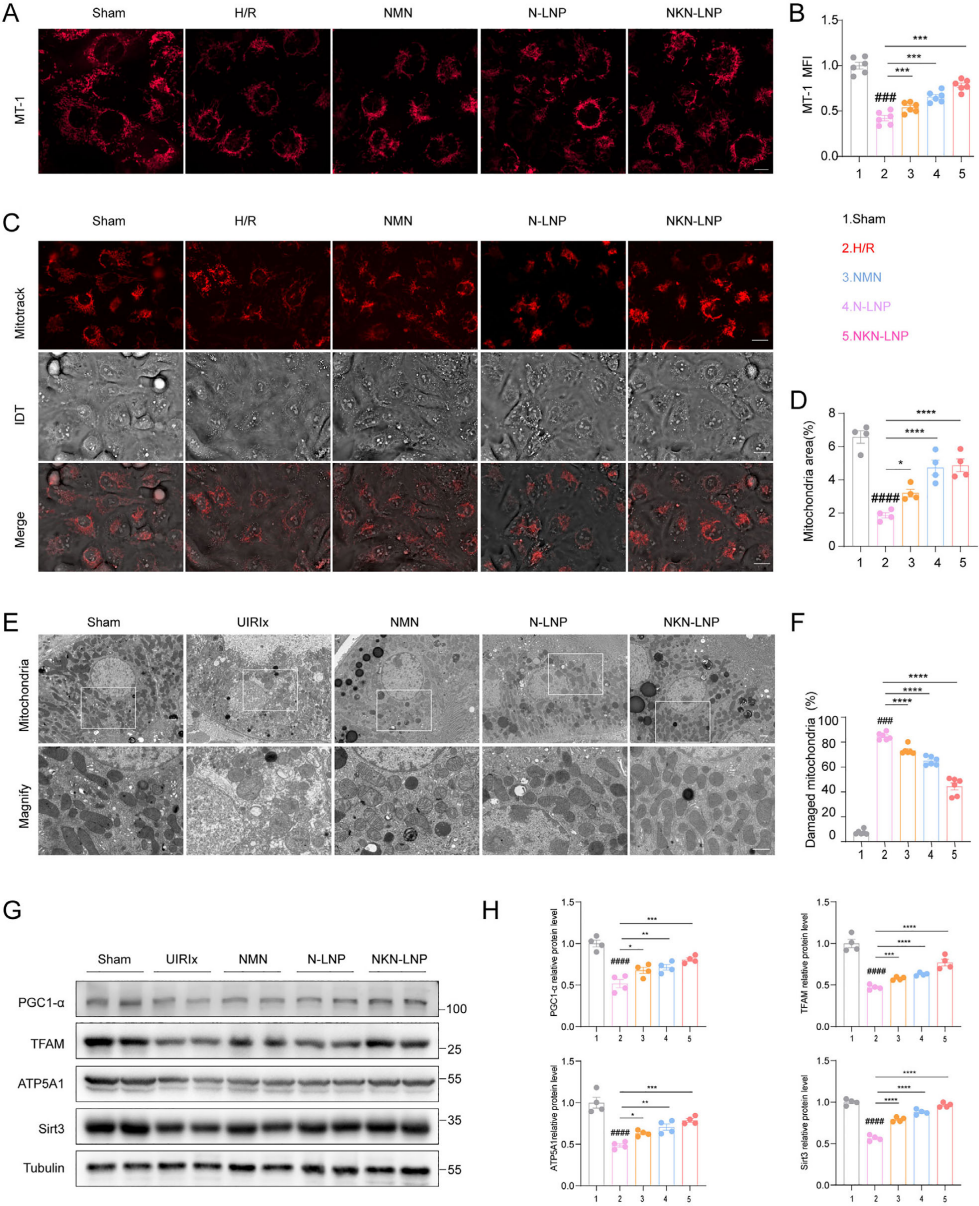
NKN‐LNP rescues mitochondrial homeostasis via SIRT3/PGC1‐α axis in AKI (A) Mitochondrial membrane potential in H/R‐treated cells following various treatments, assessed by MT‐1 staining (scale bar: 10 µm). (B) Quantification of mitochondrial membrane potential fluorescence intensity (n = 6). (C) Representative label‐free live‐cell microscopy images of H/R‐treated HK‐2 cells after MitoTracker staining under different treatment conditions (scale bar: 20 µm). (D) Quantification of mitochondrial area in (C) (n = 4 per group). (E) Representative electron microscopy images of kidney tissues from different experimental groups, illustrating mitochondrial morphology and quantity (scale bar: 1 µm). (F) Statistical analysis of damaged mitochondria as shown in panel (E) (n = 6 per group). (G) Representative Western blot images, and (H) corresponding quantification of PGC‐1α, TFAM, ATP5A1, SIRT3, and Tubulin protein levels in kidney tissues (n = 4 per group). In (B), (D), (F), (H) Data are presented as mean ± SEM. Statistical significance was determined by one‐way ANOVA followed by Tukey's post hoc test. ^*^ *p* < 0.05, ^**^ *p* < 0.01, ^***^ *p* < 0.001, ^****^ *p* < 0.0001 versus UIRIx (H/R). ^####^ *p* < 0.0001 UIRIx (H/R) versus Sham.

Transcriptomic profiling established oxidative phosphorylation (OXPHOS) regulation as the central mechanism underlying the protective effects of NKN‐LNP against kidney injury. Because mitochondria are the primary site of oxidative phosphorylation, dysfunction in mitochondrial proteins directly impairs cellular energy production. We established an in vitro hypoxia‐reoxygenation (H/R) model in HK‐2 cells to assess mitochondrial membrane potential. MT‐1 staining revealed that both N‐LNP and NKN‐LNP significantly attenuated the H/R‐induced decline in mitochondrial membrane potential (Figure 7A,B). Mitochondrial morphology and quantity were evaluated using MitoTracker staining and label‐free live‐cell microscopy (Figure 7C). Compared to the control group, H/R‐injured cells exhibited a significant reduction in mitochondrial area. Treatment with NMN, N‐LNP, or NKN‐LNP effectively restored mitochondrial density, with N‐LNP and NKN‐LNP showing superior efficacy relative to NMN (Figure 7D). We further conducted a comprehensive investigation into how NKN‐LNP influences mitochondrial integrity during kidney injury progression. High‐resolution transmission electron microscopy (TEM) examination of renal tubular epithelial cells revealed that I/R induced severe mitochondrial depletion accompanied by characteristic pathological remodeling—manifested as mitochondrial matrix swelling, cristae disorganization, and extensive vacuolization. Notably, administration of NKN‐LNP substantially restored mitochondrial abundance and mitigated structural damage, demonstrating an enhanced capacity to promote mitochondrial homeostasis under pathological conditions (Figure 7E,F). Damaged mitochondria are key sites for ROS generation. Using ROS staining on frozen kidney tissue sections, we found that NKN‐LNP most effectively reduced ROS levels in the treatment group (Figure S10A,B).

Building upon these ultrastructural observations, we investigated the molecular mechanisms underlying NKN‐LNP‐mediated mitochondrial protection. SIRT3, the primary NAD^+^‐dependent mitochondrial deacetylase, plays a critical role in regulating mitochondrial oxidative phosphorylation, redox homeostasis, and adaptive stress responses. Transcriptomic analysis demonstrated the most pronounced restoration of Sirt3 expression following treatment with NKN‐LNP (Figure S9C). Moreover, compared to the UIRIx group, NKN‐LNP treatment significantly increased the protein expression levels of SIRT3. In line with these findings, NKN‐LNP delivery notably upregulated the expression of key mitochondrial regulatory factors, including peroxisome proliferator‐activated receptor‐γ coactivator‐1α (PGC‐1α), mitochondrial transcription factor A (TFAM), and ATP synthase subunit alpha (ATP5A1) (Figure 7G,H). This effect was recapitulated in vitro, where Western blot analysis in an H/R model demonstrated that both NKN‐LNP and N‐LNP coordinately upregulated key regulators of mitochondrial function, including SIRT3, PGC‐1α, TFAM, and ATP5A1 (Figure S10C,D). Collectively, these results demonstrate that NKN‐LNP promotes mitochondrial biogenesis and functional recovery through SIRT3 activation, thereby offering protection against acute kidney injury.

### 2.7 NKN‐LNP Ameliorate Progression from Acute Kidney Injury to Chronic Kidney Disease

**FIGURE 8:**
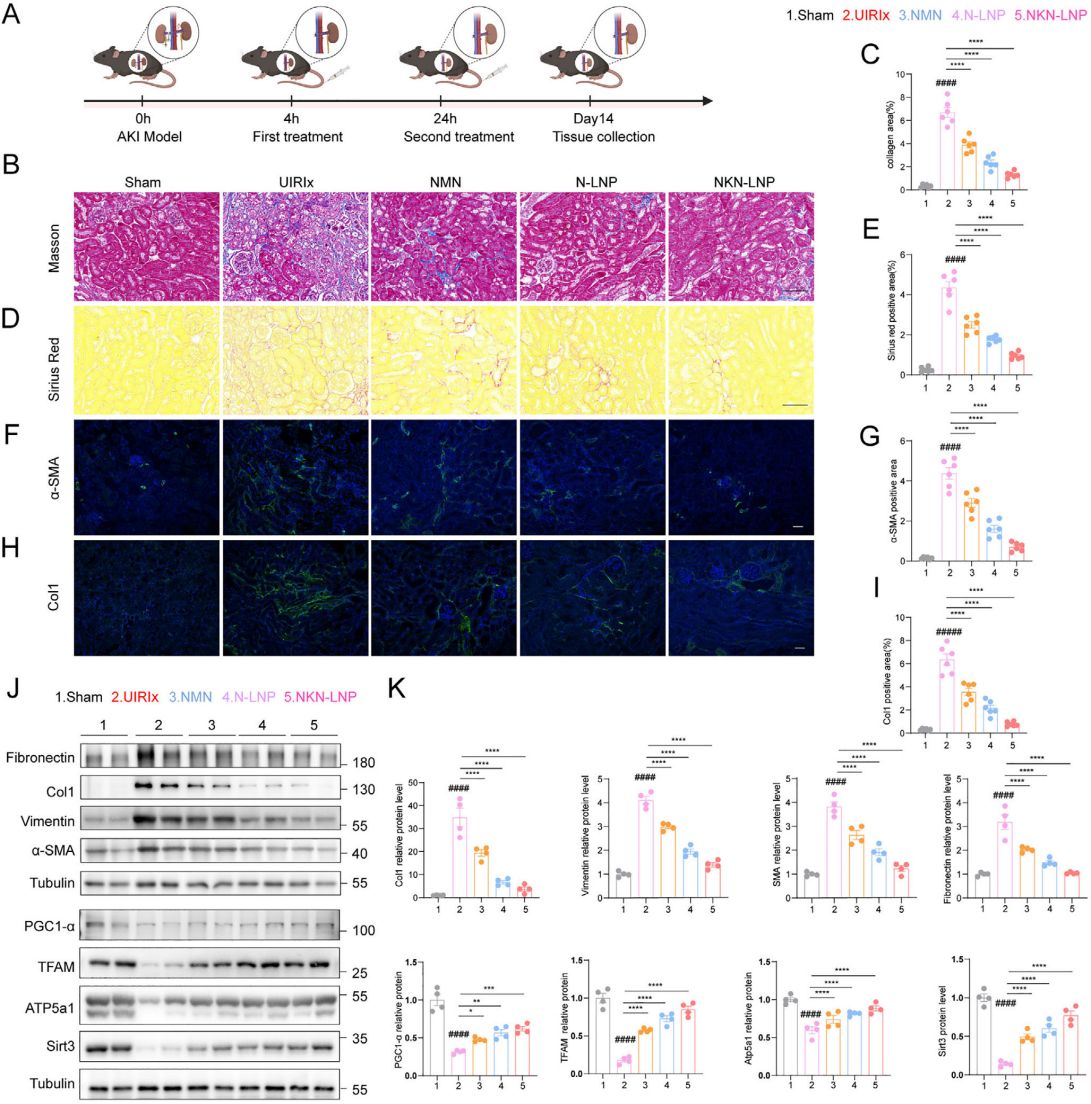
NKN‐LNP Alleviates Renal Fibrosis and Mitochondrial Dysfunction During the AKI‐to‐CKD Transition. (A) Schematic representation of the experimental protocol for the establishment of the AKI‐to‐CKD model and treatment administration. (B) Representative Masson staining images of kidney tissues from different groups (scale bar: 100 µm). (C) Quantification of collagen deposition areas based on Masson staining (n = 6). (D) Representative Sirius Red staining images of kidney tissues from each group (scale bar: 100 µm). (E) Statistical analysis of Sirius Red‐positive fibrotic areas (n = 6). (F) Representative immunofluorescence images of α‐SMA staining in kidney tissues (scale bar: 50 µm). (G) Quantification of α‐SMA‐positive areas (n = 6). (H) Representative immunofluorescence images of collagen I staining in kidney tissues (scale bar: 50 µm). (I) Statistical analysis of collagen I‐positive fibrotic areas (n = 6). (J) Representative Western blot images showing expression of α‐SMA, Vimentin, Collagen I, and Fibronectin (fibrosis markers), along with mitochondrial regulators PGC‐1α, TFAM, ATP5A1, and SIRT3 in kidney tissues across experimental groups. (K) Densitometric quantification of the proteins shown in (J) (n = 4 per group). Statistical significance in panels (C), (E), (G), (I), and (K) was evaluated using one‐way ANOVA followed by Tukey's post hoc test. Data are presented as mean ± SEM. ^*^ *p* < 0.05, ^**^ *p* < 0.01, ^***^ *p* < 0.001, ^****^ *p* < 0.0001 versus UIRIx. ^####^ *p* < 0.0001 UIRIx versus Sham.

AKI elevates the risk of CKD and end‐stage kidney disease through structural damage, inflammatory cascades, and nephron loss. To determine whether NKN‐LNP could mitigate these chronic sequelae, we developed an AKI‐to‐CKD transition model in mice. Animals received intravenous administration of saline, NMN, N‐LNP, or NKN‐LNP at 4 and 24 h post‐unilateral ischemia‐reperfusion injury (UIRIx). We harvested kidney tissues for comprehensive analysis two weeks post‐intervention (Figure 8A). Histological examination at day 14 post‐injury using Masson's trichrome and Sirius Red staining demonstrated extensive collagen deposition in saline‐treated mice, whereas NKN‐LNP treatment markedly attenuated fibrotic matrix accumulation (Figure 8B–E). Immunofluorescence analysis revealed that saline‐treated mice substantially increased expression of pro‐fibrotic markers α‐smooth muscle actin (α‐SMA) and type I collagen (COL1A1), which was significantly suppressed by NKN‐LNP treatment (Figure 8F–I). Consistent with these observations, Western blot analysis confirmed the upregulation of fibronectin, type I collagen, α‐SMA, and vimentin in the saline group, while NKN‐LNP most effectively inhibited the expression of these fibrotic proteins among the treatment groups (Figure 8J, K). These findings indicate potent anti‐fibrotic activity of the nanoparticles in preventing renal fibrosis. Mechanistically, Western blot results showed that NKN‐LNP still enhanced the protein expression of PGC‐1α, SIRT3, ATP5A1, and TFAM in the AKI‐to‐CKD model, suggesting a restoration of mitochondrial biogenesis (Figure 8J, K). Collectively, our findings demonstrate that NKN‐LNP not only ameliorate acute kidney injury but also interrupt the progression to chronic kidney disease by suppressing collagen deposition and enhancing mitochondrial function.

### 2.8 In Vivo Nanotoxicology Assessment of Engineered Nanoparticles

To evaluate nanomaterial toxicology, we implemented comprehensive in vivo safety assessments. C57BL/6J mice received intravenous NKN‐LNP or N‐LNP (8 mg/kg) every 48 h for 14 days, with saline‐treated controls. Multi‐organ toxicity assessment combined biochemical assays and histopathology. Serum hepatic markers (ALT, AST) and renal indicators (SCr, BUN) manifested no significant alterations compared to controls across treatment groups, maintaining physiological baselines (Figure S11A). Routine blood analysis showed no significant differences in WBC, RBC, or PLT among saline‐, N‐LNP‐, and NKN‐LNP–treated mice (Figure S11B). H and E‐stained sections of major organs (heart, liver, spleen, lungs, and kidneys) showed well‐preserved tissue architecture, with no signs of inflammatory infiltrates, necrosis, or pathological abnormalities in any of the cohorts (Figure S11C,D).

## 3 Conclusion

AKI and its subsequent progression to CKD represent a significant clinical challenge characterized by complex pathophysiological mechanisms and limited therapeutic options [44]. Conventional pharmacological interventions are hampered by systemic toxicity profiles and insufficient renal bioavailability [45, 46, 47]. Nanomaterial‐based delivery platforms have emerged as promising alternatives, with neutrophil‐mediated delivery systems garnering particular attention due to their inherent inflammatory tropism [48, 49, 50, 51, 52]. However, existing neutrophil‐hitchhiking strategies exhibit suboptimal efficacy owing to inadequate spatiotemporal control—nanoparticles typically remain attached to neutrophils without properly detaching at injury sites, thus failing to specifically target damaged tubular epithelium [53, 54]. We address this critical limitation through the development of a dual‐cascade responsive nanoplatform that integrates neutrophil‐guided transport with microenvironment‐triggered payload release, enabling sequential targeting of both inflammatory carriers (neutrophils) and terminal targets (KIM1^+^ tubular epithelial cells).

We demonstrate that selective targeting of KIM1 and neutrophils facilitates the efficient and sustained accumulation of encapsulated NMN in the injured kidney. Through extensive analyses, including RNA sequencing and electron microscopy, we show that this nanomaterial preserves mitochondrial function under I/R‐induced stress via the SIRT3/PGC1α pathway. This mechanism not only protects renal function but also mitigates the progression from AKI to CKD. The superior in vivo therapeutic efficacy of NKN‐LNP, despite its comparable performance to N‐LNP in the simplified H/R model in vitro, reflects delivery mechanisms that are operative only under pathological conditions. While both formulations confer direct NMN‐mediated cytoprotection in vitro, the in vivo advantage of NKN‐LNP arises from its dual‐cascade design, in which neutrophil‐mediated trafficking facilitates renal homing, followed by MMP‐2/9–triggered release at injury sites to enhance local nanoparticle accumulation and retention. This spatiotemporally regulated delivery process underlies the improved therapeutic outcomes observed in vivo. Our findings provide a solid foundation for the development of nanomedicine‐based drug delivery systems, underscoring the potential for clinical translation of NMN‐loaded nanomaterials in the treatment of AKI and following AKI‐to‐CKD. Despite these promising findings, our study has several limitations that warrant consideration. First, our research model exclusively employed ischemia‐reperfusion‐induced AKI and following AKI‐to‐CKD [55]. The efficacy of this dual‐cascade nanoplatform in treating AKI arising from other etiologies (e.g., sepsis, nephrotoxins) remains to be validated [56, 57]. Second, while the material demonstrated significant therapeutic efficacy in animal models, its translation to clinical application requires rigorous evaluation of its biosafety profile and biocompatibility in humans. Additionally, our RNA sequencing data indicated that NKN‐LNP significantly upregulated solute carrier (SLC) family transporters; however, the specific mechanisms underlying this upregulation remain unclear and warrant further investigation.

Overall, by first exploiting neutrophil‐hitchhiking and subsequently targeting renal injury molecule KIM1, this study establishes a cascade delivery platform that significantly enhances therapy for renal ischemia‐reperfusion injury.

## 4 Methods

### 4.1 Preparation of NKN‐LNP Liposome

To construct a multifunctional liposomal delivery system with dual‐targeting capabilities, we designed and synthesized the peptide KIMNEBP(CLTHVVWLPLGLAGGEAIPMSIPPEVK) containing three functional segments. This peptide integrates the neutrophil‐targeting peptide NEBP (GEAIPMSIPPEVK), MMP‐2/9‐responsive peptide segment (PLGLAG), and KIM1 targeting peptide (CLTHVVWL), achieving precise dual targeting of neutrophils and injured renal tubules. Using DSPE‐PEG2K‐Maleimide as the base material for KIMNEBP surface modification, we combined it with NMN, cholesterol, and lipid components in an organic solvent. A thin film was obtained via rotary evaporation, followed by the addition of phosphate‐buffered saline and 10‐min sonication treatment. The final NKN‐LNP nanoplatform system was isolated and purified by ultrafiltration. The liposomes were prepared using the thin‐film hydration method. Briefly, the liposome was formulated using DSPC (1,2‐distearoyl‐sn‐glycero‐3‐phosphocholine, 35%), cholesterol (35%), DOTAP (1,2‐dioleoyl‐3‐trimethylammonium‐propane (chloride), 10%), DSPE‐PEG2000 (5%), DSPE‐PEG‐NEBP (5%), and drug or dye (10%) at the indicated molar ratios.

### 4.2 Drug Loading Content and Encapsulation Efficiency

Cy5 was used as a fluorescent surrogate to evaluate the drug loading content and encapsulation efficiency of β‐nicotinamide mononucleotide in the liposome. After liposome preparation, the samples were centrifuged, and the amount of free (unencapsulated) Cy5 in the supernatant was quantified using a UV–vis spectrophotometer at 650 nm. The loading content of Cy5 in the liposome (µg mg^−^^1^) was calculated using the following equation:

(1) $$\mathrm{Loading}\;\mathrm{content}=\frac{M_{1}-M_{2}}{M_{3}}$$

where *M*_1_is the total mass of Cy5 initially added, *M*_2_is the mass of free Cy5 detected in the supernatant, and *M*_3_is the total mass of the liposome. The encapsulation efficiency (%) was calculated as:

(2) $$\mathrm{Encapsulation}\;\mathrm{efficiency}=\frac{M_{1}-M_{2}}{M_{1}}\times 100\%$$

### 4.3 Cell Culture and Treatment

The human renal tubular epithelial cell line HK‐2 (Research Resource Identifiers: 19375.09.3101HUMSCSP511) was obtained from the Cell Bank/Stem Cell Bank of the Chinese Academy of Sciences in July 2024. The cell line was confirmed to be free of mycoplasma contamination and was used at low passage numbers in all experiments. HK‐2 cells were maintained in DMEM/F12 medium. For H/R modeling, cells underwent 24 h hypoxia (94% N_2_, 1% O_2_, 5% CO_2_) in glucose/serum‐free medium followed by 2 h reoxygenation (95% air, 5% CO_2_) in complete medium. Cy5‐labeled nanoparticles were then added for 6 h of incubation for immunofluorescence analysis.

### 4.4 AKI and AKI‐to‐CKD Mouse Models

Male C57BL/6 mice (6–8 weeks old) were used for all in vivo experiments. The animals were obtained from GemPharmatech (Nanjing, China) and maintained under specific pathogen‐free (SPF) conditions. We established a unilateral ischemia‐reperfusion injury with contralateral nephrectomy (UIRIx) model following previously described protocols [55, 58]. Briefly, the renal artery and vein of the right kidney were ligated, followed by nephrectomy. The vascular pedicle of the left kidney was then occluded for 30 min before releasing the clamp to initiate reperfusion. During ischemia, the body temperature of the mice was maintained at 37°C. Mice were randomly assigned to five experimental groups (n = 6–8 per group): sham operation (Sham), UIRIx model, single‐targeted nanoparticles (N‐LNP), dual‐targeted nanoparticles (NKN‐LNP), and free drug (NMN). In the NMN treatment group, NMN (MedChemExpress, HY‐F0004, purity 99.4%) was dissolved in sterile saline and administered as described below. Treatment groups received tail vein injections 4 h post‐reperfusion (8 mg/kg). Tissue and biological samples were collected 24 h after the final dose administration.

For the AKI‐to‐CKD mouse model, UIRIx mice received intravenous administration of NMN or NKN‐LNP or N‐LNP (8 mg/kg) at 4 and 24 h post‐reperfusion. Fourteen days after injury, animals were euthanized and kidney tissues collected for histological assessment and quantification of fibrosis markers.

All animal experiments were performed in accordance with the National Institutes of Health (NIH) guidelines for the care and use of laboratory animals and were approved by the Institutional Animal Care and Use Committee of Zhejiang University (ZJU20250068).

### 4.5 In Vivo Distribution of Nanoparticles

Four hours after establishing the UIRIx model, we administered Cy5‐labeled NKN‐LNP and Cy5‐labeled N‐LNP separately via tail vein injection. Concurrently, healthy control mice received Cy5‐labeled NKN‐LNP. Major organs (liver, brain, kidney, spleen, heart, and lungs) were collected at three time points: 2, 6, and 24 h post‐administration, and subjected to fluorescence intensity detection using an in vivo imaging system (BIOSPACE LAB Photon IMAGER Optima) to systematically evaluate nanoparticle distribution across various organs. For fluorescence imaging analysis of tissue sections, we specifically labeled damaged proximal tubules with KIM1 to determine the localization relationship between nanoparticles and damaged kidney tubules.

### 4.6 Western Blot

Cells and tissues were washed with cold PBS and lysed in cold RIPA buffer containing protease and phosphatase inhibitors, followed by incubation at 4°C with horizontal shaking at 120 rpm for 30 min. The suspension was centrifuged at 12 000 rpm and 4°C for 10 min, and protein concentration in the supernatant was determined using a BCA assay kit (Beyotime, Shanghai, China). Samples were adjusted to a final concentration of 5 mg/mL total protein, boiled at 95°C for 10 min, and centrifuged at 12 000 rpm for 1 min. Equal amounts of protein and appropriate molecular weight markers were loaded into SDS‐PAGE gel wells and electrophoresed at 100 V for 70 min. Proteins were transferred to PVDF membranes using wet transfer at 120 V for 90 min. The PVDF membranes were blocked in 5% non‐fat milk (blocking buffer) for 1 h at room temperature, then incubated with primary antibodies (Table S1) overnight at 4°C with horizontal agitation. Membranes were washed three times with TBST for 5 min each, followed by incubation with secondary antibodies (Table S1) diluted in blocking buffer for 1 h at room temperature. After repeated TBST washing, the secondary antibody signals were detected using a Tanon 5200 imaging system.

### 4.7 Isolation of Neutrophils from Mouse Bone Marrow

Neutrophils were isolated from the bone marrow of 8‐ to 10‐week‐old C57BL/6 mice. Under aseptic conditions, femurs and tibias were dissected and placed in sterile centrifuge tubes. The bones were then centrifuged at 8000 rpm in a pre‐cooled centrifuge to collect bone marrow cells. The cell suspension was subjected to density gradient centrifugation using a separation kit (TBDsciences, TBD2013NM) according to the manufacturer's protocol.

Cell purity (> 90%) was confirmed by flow cytometry using anti‐CD11b‐APC (Cell Signaling Technology, #41249) and anti‐Ly6G‐FITC (Cell Signaling Technology, #88876). Isolated neutrophils were resuspended in high‐glucose DMEM medium (Basalmedia, Shanghai, China) supplemented with 10% FBS and 1% penicillin‐streptomycin (Basalmedia, Shanghai, China) and maintained at 37°C in a 5% CO_2_ incubator for subsequent experiments. The gating strategy for identifying neutrophils (CD11b^+^ Ly6G^+^) is detailed in Figure S1D (peripheral blood) and Figure S6 (bone marrow).

### 4.8 Detection of Mitochondrial Membrane Potential

Following treatment, cells from each experimental group were washed twice with pre‐warmed Hanks’ Balanced Salt Solution (HBSS; Sangon Biotech, A003210) to remove residual medium. The mitochondrial membrane potential was assessed using the MT‐1 fluorescent dye (Mitochondrial Membrane Potential Assay Kit, DOJINDO, #MT13) according to the manufacturer's protocol. Briefly, the MT‐1 staining solution was diluted 1:1000 in serum‐free culture medium and applied to the cells in a confocal dish. After incubation at 37°C with 5% CO_2_ for 30 min, unbound dye was removed by washing the cells three times with HBSS. Then live‐cell imaging was performed immediately using a confocal laser scanning microscope (IXplore SpinSR Olympus) equipped with a 100× oil‐immersion objective. For quantitative analysis, fluorescence intensity was measured using Fiji (ImageJ).

### 4.9 Label‐Free Live Cell Microscopy System

In this study, a label‐free live cell microscopy system (SC3000; Zircon Optoelectronics, Suzhou, China) was employed to observe biological samples. The system's core imaging principle is based on intensity diffraction tomography (IDT). Upon illumination with an incident beam, transparent biological specimens—such as live cells—induce wavefront distortions in the transmitted light field due to localized differences in subcellular refractive index (RI).

### 4.10 Cellular and Tissue Immunofluorescence Staining

Cells were seeded onto cell culture coverslips placed in 24‐well plates and allowed to adhere. After attachment, cells were subjected to different experimental treatments. Following treatment, cells were fixed with 4% paraformaldehyde at room temperature for 15 min. After washing with PBS, nonspecific binding sites were blocked with 10% normal goat serum in PBS and incubated for 1 h at room temperature. Primary antibodies (Table S1) were diluted in blocking solution and incubated with samples at 4°C overnight. After three 5‐min PBS washes, cells were incubated with species‐matched Alexa Fluor‐conjugated secondary antibodies and 4,6‐diamidino‐2‐phenylindole (DAPI; YEASEN) (Table S1) for 1 h at room temperature under light‐protected conditions. Fluorescence images were acquired using a [IXplore SpinSR Olympus] laser scanning confocal microscope. Fluorescence intensity quantification was analyzed using Fiji (ImageJ).

Fresh kidney tissue samples were fixed in 4% paraformaldehyde at 4°C overnight, followed by gradient dehydration in 20% and 30% sucrose solutions. After dehydration, the tissues were embedded in OCT compound and sectioned into 10‐µm thick slices. The immunofluorescence staining steps were consistent with those used for cell immunofluorescence.

### 4.11 Immunohistochemical Analysis

Kidney tissues were fixed in 4% paraformaldehyde at 4°C overnight, embedded in paraffin, and sectioned into 6‐µm‐thick transverse slices. Immunohistochemical staining was performed according to the instructions provided by the immunohistochemistry kit (Haoke).

### 4.12 Renal Function and Histological Examination

We quantified serum creatinine (SCr), blood urea nitrogen (BUN), alanine aminotransferase (ALT), and aspartate aminotransferase (AST) with commercial assay kits (FUJFILM). Kidney specimens were fixed in 4% paraformaldehyde, embedded in paraffin, and sectioned for histological analysis. We performed H&E, Masson's trichrome, and Sirius Red staining following the protocols provided by (Servicebio China). Renal tubular injury was assessed using a semi‐quantitative scale (0–5), which considered the degree of necrotic epithelium, luminal debris, cast formation, tubular dilation, and epithelial desquamation. The scoring system was based on the percentage of affected tubules: 0 (minimal, < 5%), 1 (mild, 5–10%), 2 (moderate, 11–25%), 3 (marked, 26–45%), 4 (severe, 46–75%), and 5 (extensive, > 76%), based on previous reports [59].

### 4.13 NAD^+^ Quantification

Renal tissues were homogenized on ice using NAD^+^/NADH Extraction Buffer (Beyotime, Shanghai, China, S0175‐5) and centrifuged at 12 000 × g for 10 min at 4°C. Supernatant absorbance at 450 nm was measured via a microplate reader. Total NAD was quantified using a dehydrogenase‐coupled enzymatic assay based on the reduction of WST‐8 to formazan, following the manufacturer's instructions. And NAD^+^ concentrations were calculated against a standard curve prepared with NAD^+^ reference material.

### 4.14 Transcriptomics Analysis

RNA was extracted from the renal cortex using TRIzol reagent (Sangon Biotech, Shanghai, China) and subsequently processed for mRNA sequencing and data analysis by Hangzhou Cosmos Wisdom Biotech Co., Ltd. The sequencing data have been deposited in the National Center for Biotechnology Information (NCBI) Sequence Read Archive (SRA) under accession number PRJNA1431578.

### 4.15 Bioinformatics Analysis

Murine renal scRNA‐seq dataset (GSE274819) was retrieved from the NCBI GEO repository (https://www.ncbi.nlm.nih.gov/geo/query/acc.cgi?acc=GSE274819) [38]. Analysis was conducted in R (v4.2.1) using the Seurat package (v4.2.1). Cells were filtered based on standard quality control metrics: retaining cells with more than 200 and fewer than 2500 detected genes (nFeature_RNA) and less than 10% mitochondrial gene expression (percent.mt). The R package Harmony was used to correct for batch effects between samples.

### 4.16 Patient Samples Collection

We enrolled twenty‐nine patients undergoing partial nephrectomy at the Fourth Affiliated Hospital of Zhejiang University, from whom complete blood counts were obtained before and after surgery (Table S2). All participants provided written informed consent. The study protocol was approved by The Fourth Affiliated Hospital of Zhejiang University School of Medicine Human Research Ethics Committee (K2025098) and conducted in accordance with the principles of the Declaration of Helsinki.

### 4.17 Statistical Analysis

Statistical analyses were performed using GraphPad Prism 8.0.2 (GraphPad Software, Inc.). All data are presented as mean ± SEM, unless otherwise indicated. For non‐transcriptomic data, no data transformation or normalization was applied prior to statistical analysis. Data were assessed for normality using the Shapiro–Wilk test, and no data points were excluded as outliers unless explicitly stated. Sample sizes (n) represent the number of independent biological replicates and are specified in the corresponding figure legends. *p* value < 0.05 was considered statistically significant. Significance was defined as follows in the figure legends: ^*^ *p* < 0.05, ^**^ *p* < 0.01, ^***^ *p* < 0.001, or ^****^ *p* < 0.0001. The data were first checked for normality. When comparing two groups, a two‐tailed unpaired Student's t‐test was used if the data were normally distributed, and variances were similar (confirmed via an F‐test, *p* > 0.05). If variances differed (*p* < 0.05), Welch's correction was applied. For non‐normally distributed data, a Mann‐Whitney test was employed. Comparisons of paired data were performed using a two‐tailed paired Student's t‐test. For comparisons among three or more groups, one‐way ANOVA followed by Tukey's post hoc test was used.

## Conflicts of Interest

The authors declare no conflict of interest.

## Supporting information




**Supporting File 1**: advs74824‐sup‐0001‐SuppMat.docx.

**Supporting File 2**: advs74824‐sup‐0002‐FigureS1‐S11.zip.

**Supporting File 3**: advs74824‐sup‐0003‐TableS1‐S2.docx.

## Data Availability

The data that support the findings of this study are available from the corresponding author upon reasonable request.

## References

[1] M. Ostermann , N. Lumlertgul , R. Jeong , E. See , M. Joannidis , and M. James , “Acute Kidney Injury,” Lancet 405 (2025): 241–256, 10.1016/s0140-6736(24)02385-7. 39826969

[2] F. Turgut , A. S. Awadand , and E. M. Abdel‐Rahman , “Acute Kidney Injury: Medical Causes and Pathogenesis,” Journal of Clinical Medicine 12 (2023): 375, 10.3390/jcm12010375. 36615175 PMC9821234

[3] A. Vijayan , “Tackling AKI: Prevention, Timing of Dialysis and Follow‐up,” Nature Reviews Nephrology 17 (2021): 87–88, 10.1038/s41581-020-00390-3. 33335277 PMC7745587

[4] J. A. Kellum , P. Romagnani , G. Ashuntantang , C. Ronco , A. Zarbock , and H.‐J. Anders , “Acute Kidney Injury,” Nature Reviews Disease Primers 7 (2021): 52, 10.1038/s41572-021-00284-z. 34267223

[5] T. Zhang , R. E. Widdopand , and S. D. Ricardo , “Transition from Acute Kidney Injury to Chronic Kidney Disease: Mechanisms, Models, and Biomarkers,” American Journal of Physiology‐Renal Physiology 327 (2024): F788–F805, 10.1152/ajprenal.00184.2024. 39298548

[6] M. D. Okusa , M. H. Rosner , J. A. Kellumand , and C. Ronco , “Therapeutic Targets of Human AKI,” Journal of the American Society of Nephrology 27 (2016): 44–48, 10.1681/asn.2015030233. 26519086 PMC4696586

[7] B. C. Birkelo , J. L. Koyner , M. Ostermannand , and P. K. Bhatraju , “The Road to Precision Medicine for Acute Kidney Injury,” Critical Care Medicine 52 (2024): 1127–1137, 10.1097/ccm.0000000000006328. 38869385 PMC11250999

[8] R. Matsuura , K. Doiand , and H. Rabb , “Acute Kidney Injury and Distant Organ Dysfunction–Network System Analysis,” Kidney International 103 (2023): 1041–1055, 10.1016/j.kint.2023.03.025. 37030663

[9] P. Li , J. Liu , and Y. Wang , “Spatiotemporal Targeted Delivery of Biomimetic Bacterial Outer Membrane Nanoparticles for Enhanced Spinal Cord Injury Repair,” Advanced Materials 37 (2025): 2502795, 10.1002/adma.202502795. 40391641

[10] Q. Mu , K. Yao , and M. Z. Syeda , “Neutrophil Targeting Platform Reduces Neutrophil Extracellular Traps for Improved Traumatic Brain Injury and Stroke Theranostics,” Advanced Science 11 (2024): 2308719, 10.1002/advs.202308719. 38520727 PMC11151022

[11] M. Zhou , Y. Tang , and Y. Lu , “Framework Nucleic Acid‐Based and Neutrophil‐Based Nanoplatform Loading Baicalin with Targeted Drug Delivery for Anti‐Inflammation Treatment,” ACS Nano 19 (2025): 3455–3469, 10.1021/acsnano.4c12917. 39817852

[12] Q. Mu , K. Yao , and M. Z. Syeda , “Ligustrazine Nanoparticle Hitchhiking on Neutrophils for Enhanced Therapy of Cerebral Ischemia‐Reperfusion Injury,” Advanced Science 10 (2023): 2301348, 10.1002/advs.202301348. 37078794 PMC10323616

[13] L. Tang , Z. Wang , and Q. Mu , “Targeting Neutrophils for Enhanced Cancer Theranostics,” Advanced Materials 32 (2020): 2002739, 10.1002/adma.202002739. 32656801

[14] H.‐X. Yuan , Y. Y. Ye , and P. Shen , “Engineering Neutrophil Vesicles for Synergistic Protection against Ischemia/Reperfusion Injury after Lung Transplant,” Advanced Science 12 (2025): 06127, 10.1002/advs.202506127. PMC12622495 40810721

[15] P. Shen , K. Huang , and X. Zhang , “Genetically Engineered MSC‐derived Hybrid Cellular Vesicles for ROS‐scavenging and Mitochondrial Homeostasis in Hepatic Ischemia‐reperfusion Injury,” Materials Today Bio 34 (2025): 102215, 10.1016/j.mtbio.2025.102215. PMC12398874 40893375

[16] K. Lee , H. R. Jangand , and H. Rabb , “Lymphocytes and Innate Immune Cells in Acute Kidney Injury and Repair,” Nature Reviews Nephrology 20 (2024): 789–805, 10.1038/s41581-024-00875-5. 39095505

[17] Y. Pan , S. Cao , and Y. Wang , “Myeloid EGFR Deficiency Accelerates Recovery from AKI via Macrophage Efferocytosis and Neutrophil Apoptosis,” Nature Communications 16 (2025): 4563, 10.1038/s41467-025-59393-y. PMC12084582 40379634

[18] M. J. Kimlinger , T. J. No , and E. H. Mace , “Hyperoxia Increases Kidney Injury during Renal Ischemia and Reperfusion in Mice,” Anesthesia and Analgesia 137 (2023): 996–1006, 10.1213/ane.0000000000006600. 37678264 PMC10592523

[19] A. Lau , J. J. Rahn , and M. Chappellaz , “Dipeptidase‐1 Governs Renal Inflammation during Ischemia Reperfusion Injury,” Science Advances 8 (2022): abm0142, 10.1126/sciadv.abm0142. PMC8809686 35108057

[20] R. M. Ferreira , A. R. Sabo , and S. Winfree , “Integration of Spatial and Single‐cell Transcriptomics Localizes Epithelial Cell–immune Cross‐talk in Kidney Injury,” JCI Insight 6 (2021), 10.1172/jci.insight.147703. PMC8262485 34003797

[21] Y. Yang , J. Du , and J. Gan , “Neutrophil‐Mediated Nanozyme Delivery System for Acute Kidney Injury Therapy,” Advanced Healthcare Materials 13 (2024): 2401198, 10.1002/adhm.202401198. 38899383

[22] C. Ding , B. Wang , and J. Zheng , “Neutrophil Membrane‐Inspired Nanorobots Act as Antioxidants Ameliorate Ischemia Reperfusion‐Induced Acute Kidney Injury,” ACS Applied Materials & Interfaces 15 (2023): 40292–40303, 10.1021/acsami.3c08573. 37603686

[23] R. Mathur , S. Elsafy , and A. T. Press , “Neutrophil Hitchhiking Enhances Liposomal Dexamethasone Therapy of Sepsis,” ACS Nano 18 (2024): 28866–28880, 10.1021/acsnano.4c09054. 39393087

[24] J. Pan , Z. Wang , and X. Huang , “Bacteria‐Derived Outer‐Membrane Vesicles Hitchhike Neutrophils to Enhance Ischemic Stroke Therapy,” Advanced Materials 35 (2023): 2301779, 10.1002/adma.202301779. 37358255

[25] M. Fontecha‐Barriuso , A. M. Lopez‐Diaz , S. Carriazo , A. Ortizand , and A. B. Sanz , “Nicotinamide and Acute Kidney Injury,” Clinical Kidney Journal 14 (2021): 2453–2462, 10.1093/ckj/sfab173. 34950458 PMC8690056

[26] A. I. Mede , G. L. Milne , D. Wei , D. K. Smithand , and L. E. Smith , “NAD+ Biosynthesis Impairment and Acute Kidney Injury after Major Vascular Surgery,” Antioxidants (Basel) 12 (2023): 821, 10.3390/antiox12040821. 37107195 PMC10135380

[27] M. Morevati , E. F. Fang , and M. L. Mace , “Roles of NAD+ in Acute and Chronic Kidney Diseases,” International Journal of Molecular Sciences 24 (2022): 137, 10.3390/ijms24010137. 36613582 PMC9820289

[28] R. Alhumaidi , H. Huang , M. C. Saade , A. J. Clarkand , and S. M. Parikh , “NAD+ Metabolism in Acute Kidney Injury and Chronic Kidney Disease Transition,” Trends in Molecular Medicine 31 (2025): 669–681, 10.1016/j.molmed.2024.12.004. 39757045 PMC12226734

[29] M. Bugarski , S. Ghazi , M. Polesel , J. R. Martinsand , and A. M. Hall , “Changes in NAD and Lipid Metabolism Drive Acidosis‐Induced Acute Kidney Injury,” Journal of the American Society of Nephrology 32 (2021): 342–356, 10.1681/asn.2020071003. 33478973 PMC8054907

[30] Y. Lei , Y. Wu , and W.‐R. Zhuang , “NAD+ Biosynthesis and Mitochondrial Repair in Acute Kidney Injury via Ultrasound‐responsive Thylakoid‐integrating Liposomes,” Nature Biomedical Engineering 9 (2025): 1740–1757, 10.1038/s41551-025-01402-y. 40461655

[31] N. Braidy , J. Berg , and J. Clement , “Role of Nicotinamide Adenine Dinucleotide and Related Precursors as Therapeutic Targets for Age‐Related Degenerative Diseases: Rationale, Biochemistry, Pharmacokinetics, and Outcomes,” Antioxidants and Redox Signaling 30 (2019): 251–294, 10.1089/ars.2017.7269. 29634344 PMC6277084

[32] J. Yoshino , J. A. Baurand , and S. I. Imai , “NAD+ Intermediates: The Biology and Therapeutic Potential of NMN and NR,” Cell Metabolism 27 (2018): 513–528, 10.1016/j.cmet.2017.11.002. 29249689 PMC5842119

[33] R. Duan , Y. Li , and R. Zhang , “Reversing Acute Kidney Injury through Coordinated Interplay of Anti‐Inflammation and Iron Supplementation,” Advanced Materials 35 (2023): 2301283, 10.1002/adma.202301283. 37029662

[34] Y. Kong , X. Chen , and F. Liu , “Ultrasmall Polyphenol‐NAD + Nanoparticle‐Mediated Renal Delivery for Mitochondrial Repair and Anti‐Inflammatory Treatment of AKI‐to‐CKD Progression,” Advanced Materials 36 (2024): 2310731, 10.1002/adma.202310731. 38805174

[35] S. Bengatta , C. Arnould , and E. Letavernier , “MMP9 and SCF Protect from Apoptosis in Acute Kidney Injury,” Journal of the American Society of Nephrology 20 (2009): 787–797, 10.1681/asn.2008050515. 19329763 PMC2663840

[36] G. Guvercin , V. Karakus , and M. Aksit , “Matrix metalloproteinase‐9, 10, and Stress Hyperglycaemia in Acute Kidney Injury,” European Journal of Clinical Investigation 48 (2018): 12963, 10.1111/eci.12963. 29856477

[37] D. Shen , L. Lin , and Y. Su , “Inhibition of Matrix Metalloproteinase‐9 Attenuates Kidney Fibrosis and Cellular Senescence in the Transition from Acute Kidney Injury to Chronic Kidney Disease,” Renal Failure 47 (2025): 2499897, 10.1080/0886022x.2025.2499897. 40468751 PMC12143010

[38] Y. Li , Z. Wang , and H. Xu , “Targeting the Transmembrane Cytokine co‐receptor Neuropilin‐1 in Distal Tubules Improves Renal Injury and Fibrosis,” Nature Communications 15 (2024): 5731, 10.1038/s41467-024-50121-6. PMC11231174 38977708

[39] A. Suzuki , S. A. Stern , and O. Bozdagi , “Astrocyte‐neuron Lactate Transport Is Required for Long‐term Memory Formation,” Cell 144 (2011): 810–823, 10.1016/j.cell.2011.02.018. 21376239 PMC3073831

[40] K. T. Bush , W. Wu , C. Lunand , and S. K. Nigam , “The Drug Transporter OAT3 (SLC22A8) and Endogenous Metabolite Communication via the Gut–liver–Kidney Axis,” Journal of Biological Chemistry 292 (2017): 15789–15803, 10.1074/jbc.M117.796516. 28765282 PMC5612110

[41] S. K. Nigam , “The SLC22 Transporter Family: A Paradigm for the Impact of Drug Transporters on Metabolic Pathways, Signaling, and Disease,” Annual Review of Pharmacology and Toxicology 58 (2018): 663–687, 10.1146/annurev-pharmtox-010617-052713. PMC6225997 29309257

[42] S. K. Nigam , W. Wu , K. T. Bush , M. P. Hoenig , R. C. Blantz , and V. Bhatnagar , “Handling of Drugs, Metabolites, and Uremic Toxins by Kidney Proximal Tubule Drug Transporters,” Clinical Journal of the American Society of Nephrology 10 (2015): 2039–2049, 10.2215/cjn.02440314. 26490509 PMC4633783

[43] S. K. Nigam and V. Bhatnagar , “The Systems Biology of Uric Acid Transporters,” Current Opinion in Nephrology and Hypertension 27 (2018): 305–313, 10.1097/mnh.0000000000000427. 29847376 PMC6275126

[44] K. Fukazawa and H. T. Lee , “Volatile Anesthetics and AKI,” Journal of the American Society of Nephrology 25 (2014): 884–892, 10.1681/asn.2013111215. 24511126 PMC4005317

[45] M. Meersch , T. Mayerhöferand , and M. Joannidis , “Acute Kidney Injury Subphenotyping and Personalized Medicine,” Current Opinion in Critical Care 30 (2024): 555–562, 10.1097/mcc.0000000000001212. 39503205

[46] G. Hariri and M. Legrand , “New Drugs for Acute Kidney Injury,” Journal of Intensive Medicine 5 (2025): 3–11, 10.1016/j.jointm.2024.08.001. 39872831 PMC11763585

[47] A. Messina , M. Calatroni , G. Castellani , S. De Rosa , M. Ostermann , and M. Cecconi , “Understanding Fluid Dynamics and Renal Perfusion in Acute Kidney Injury Management,” Journal of Clinical Monitoring and Computing 39 (2025): 73–83, 10.1007/s10877-024-01209-3. 39198361

[48] A. Roointan , R. Xu , S. Corrie , C. E. Hagemeyerand , and K. Alt , “Nanotherapeutics in Kidney Disease,” Journal of the American Society of Nephrology 36 (2025): 500–518, 10.1681/asn.0000000608. 39705082 PMC11888965

[49] S. Keshavan , P. Calligari , L. Stella , L. Fusco , L. G. Delogu , and B. Fadeel , “Nano‐bio Interactions: A Neutrophil‐centric View,” Cell Death & Disease 10 (2019): 569, 10.1038/s41419-019-1806-8. 31358731 PMC6662811

[50] H. Wang , J. Zang , Z. Zhao , Q. Zhangand , and S. Chen , “The Advances of Neutrophil‐Derived Effective Drug Delivery Systems: A Key Review of Managing Tumors and Inflammation,” International Journal of Nanomedicine 16 (2021): 7663–7681, 10.2147/ijn.S328705. 34815670 PMC8605828

[51] H. Wang , L. Li , L. Luo , et al., “Advanced Nanomaterial Platforms for Targeted Therapy of Myocardial Ischemia‐Reperfusion Injury,” Research 8 (2025): 0822, 10.34133/research.0822. 40765995 PMC12324810

[52] Y. Zhang , J. Wu , and Q. An , “Renal Tubule‐targeted Dexrazoxane Suppresses Ferroptosis in Acute Kidney Injury by Inhibiting ACMSD,” Nano Research 16 (2023): 9701–9714, 10.1007/s12274-023-5547-8.

[53] S. Sun , W. Lv , S. Li , et al., “Smart Liposomal Nanocarrier Enhanced the Treatment of Ischemic Stroke through Neutrophil Extracellular Traps and Cyclic Guanosine Monophosphate‐Adenosine Monophosphate Synthase‐Stimulator of Interferon Genes (cGAS‐STING) Pathway Inhibition of Ischemic Penumbra,” ACS Nano 17 (2023): 17845–17857, 10.1021/acsnano.3c03390. 37712845

[54] N. Vij , T. Min , M. Bodas , A. Gordeand , and I. Roy , “Neutrophil Targeted Nano‐drug Delivery System for Chronic Obstructive Lung Diseases,” Nanomedicine: Nanotechnology, Biology and Medicine 12 (2016): 2415–2427, 10.1016/j.nano.2016.06.008. 27381067

[55] Y. Fu , Y. Xiang , Q. Wei , D. Ilatovskayaand , and Z. Dong , “Rodent Models of AKI and AKI‐CKD Transition: An Update in 2024,” American Journal of Physiology‐Renal Physiology 326 (2024): F563–F583, 10.1152/ajprenal.00402.2023. 38299215 PMC11208034

[56] S. S. Motwani , S. S. Kaurand , and A. Kitchlu , “Cisplatin Nephrotoxicity: Novel Insights into Mechanisms and Preventative Strategies,” Seminars in Nephrology 42 (2022): 151341, 10.1016/j.semnephrol.2023.151341. 37182407

[57] T. Pais , S. Jorgeand , and J. A. Lopes , “Acute Kidney Injury in Sepsis,” International Journal of Molecular Sciences 25 (2024): 5924, 10.3390/ijms25115924. 38892111 PMC11172431

[58] J. Miao , H. Zhu , J. Wang , J. Chen , F. Han , and W. Lin , “Experimental Models for Preclinical Research in Kidney Disease,” Zoological Research 45 (2024): 1161–1174, 10.24272/j.issn.2095-8137.2024.072. 39257378 PMC11491777

[59] M. Zhou , W. Tang , and Y. Fu , “Progranulin Protects against Renal Ischemia/Reperfusion Injury in Mice,” Kidney International 87 (2015): 918–929, 10.1038/ki.2014.403. 25607110

