# Supplementary material for: Neutrophil‐Driven Cascade‐Targeted Nanocarriers Restore Mitochondrial Homeostasis to Ameliorate Renal Ischemia–Reperfusion Injury
Source: Adv Sci (Weinh). 2026 Mar 30;13(30):e20940. doi: 10.1002/advs.202520940 (PMC13248797; doi:10.1002/advs.202520940)
Supplement: Supplementary file 1 — Supporting File 1: advs74824‐sup‐0001‐SuppMat.docx. [file ADVS-13-e20940-s003.docx]

**Supporting Information**

**Neutrophil-Driven Cascade-Targeted Nanocarriers Restore Mitochondrial Homeostasis to Ameliorate Renal Ischemia–Reperfusion Injury**

*Hangbin Ma^#^, Yang Li^#^, Shen Lin^#^, Feifan Chu, Gaozhan Ren, Yinhui Mao, Mingzhi Wu, Yuning Ma, Qiwei Ji, Zujie Chen, Jinzhong Ji, Mingxin Sun, Yongpeng Xu, Xiaoli Sun *, Longguang Tang *, Hao Zhou**

HB. M, S. L, FF. C, GZ. R, YH. M, MZ. W, YN. M, QW. J, ZJ. C, MX. S, YP. X,

H. Z

Department of Urology, Center for Reproductive Medicine, The Fourth Affiliated Hospital of School of Medicine and International School of Medicine, International Institutes of Medicine, Zhejiang University, Yiwu 322000, China

E-mail: [haozhou@zju.edu.cn](mailto:haozhou@zju.edu.cn) (H. Z)

Y. L, XL. S, LG. T

Department of Pharmacy, Center for Regenerative and Aging Medicine, The Fourth Affiliated Hospital of School of Medicine and International School of Medicine, International Institutes of Medicine, Zhejiang University, Yiwu 322000, China

E-mail: [tanglongguang@zju.edu.cn](mailto:tanglongguang@zju.edu.cn) (LG. T); [xl.sun@zju.edu.cn](mailto:xl.sun@zju.edu.cn) (XL. S)
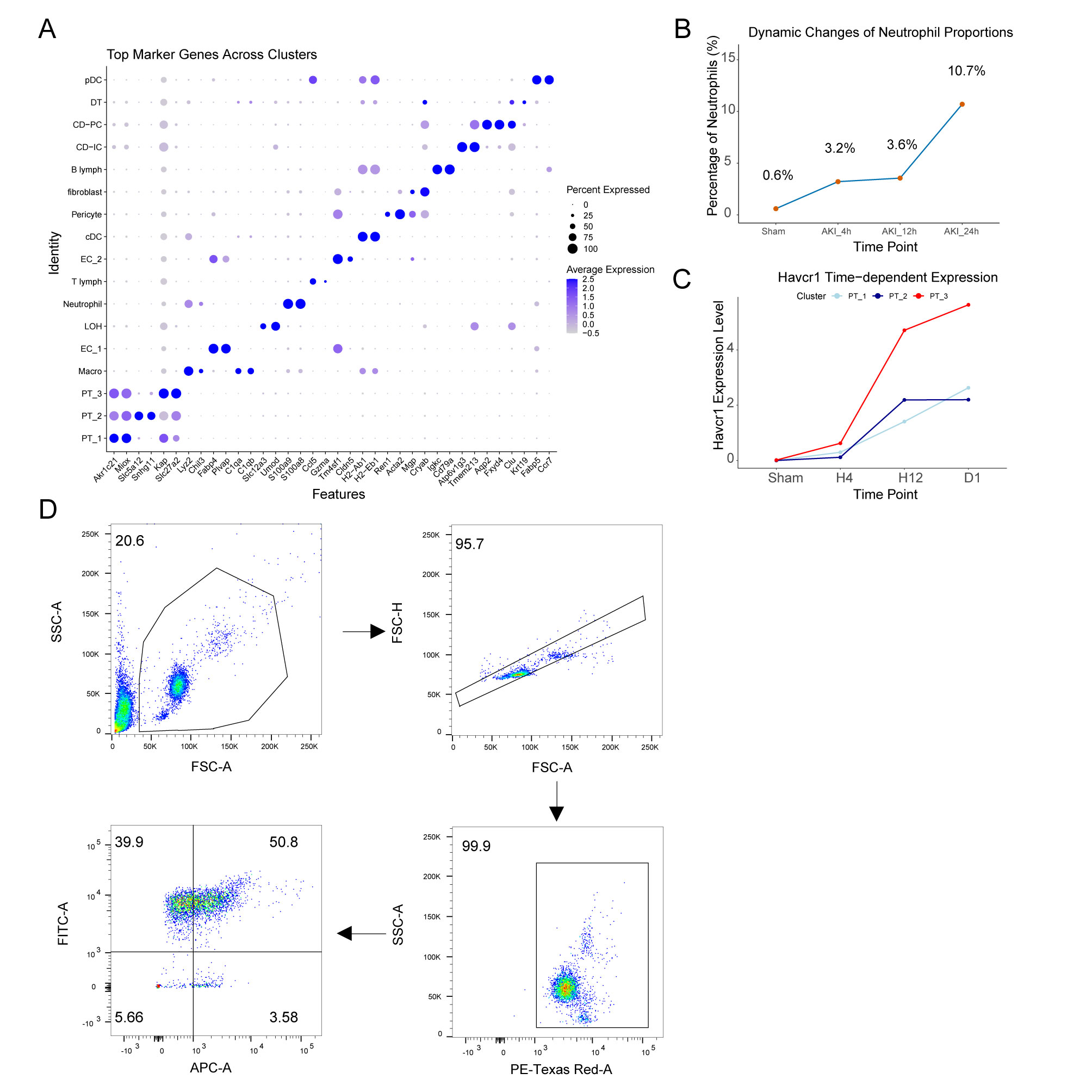


**Figure S1.** Temporal dynamics of neutrophil infiltration and injury progression in AKI

(A) Single-cell clustering analysis of Ischemic Kidney Injury Tissues (GSE274819) with cell-type annotation based on canonical markers. (B) Proportional increase of neutrophils (Ly6G^+^ cells) over time post-injury (0 h, 4 h, 12 h, and 24 h). (C) Upregulation of tubular injury marker Havcr1 (KIM1) in renal epithelial cells during AKI progression (0 h,4 h,12 h, and 24 h). (D) Gating strategy for neutrophils from UIRIx mouse peripheral blood. Sequential gating on forward and side scatter (FSC/SSC) was performed to identify granulocytes, followed by the isolation of CD11b⁺Ly6G⁺ neutrophils. Numbers indicate the percentage of cells within the respective gate.


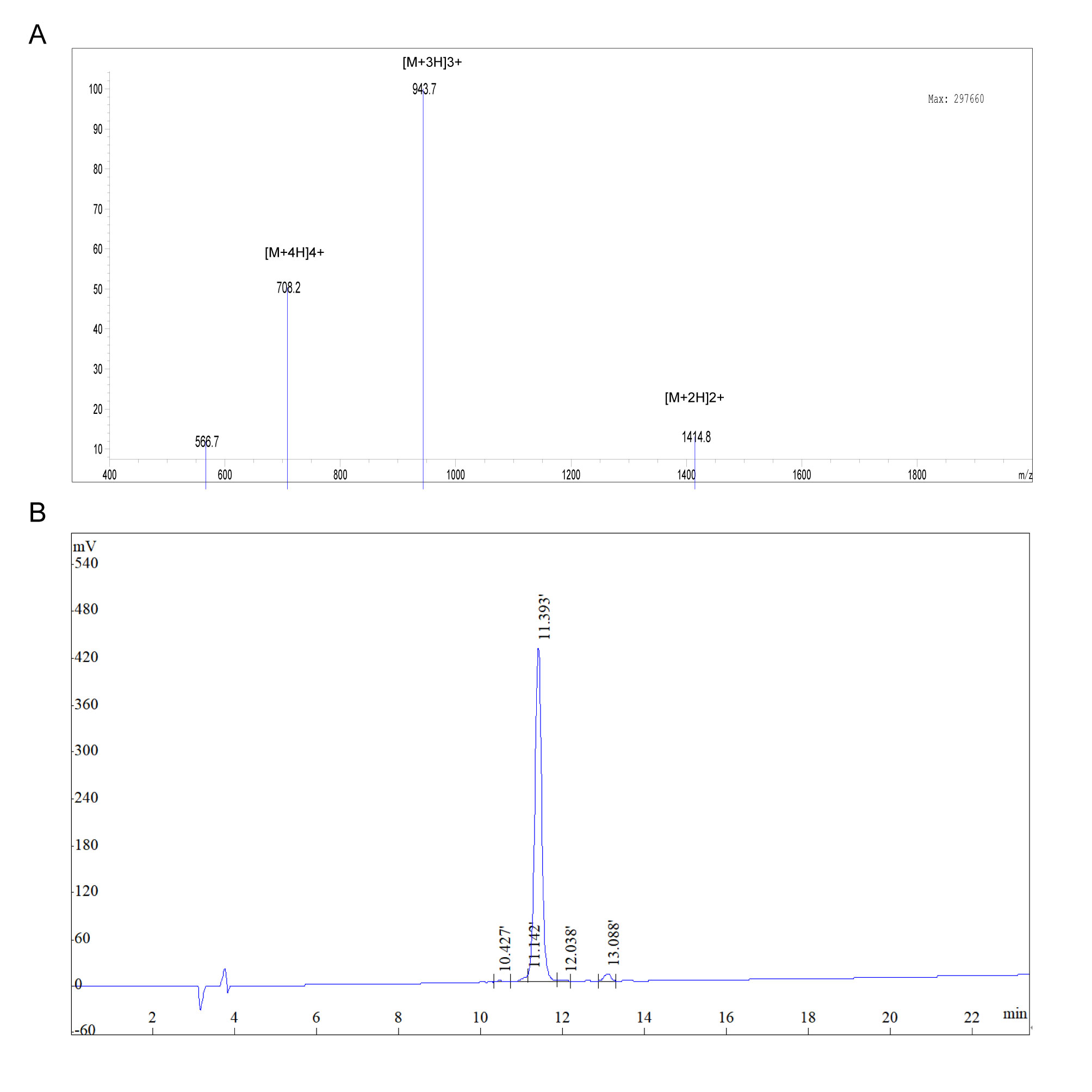


**Figure S2.** Structural confirmation and purity analysis of peptide KIMNEBP (Sequence: CLTHVVWLPLGLAGGEAIPMSIPPEVK). (A) ESI-MS analysis of the peptide was performed in positive ion mode. HRMS (m/z) calcd for KIMNEBP: 2828.39. The spectrum shows a series of multiply-charged ions, including [M+2H]²⁺, [M+3H]³⁺, and [M+4H]⁴⁺. The observed m/z values for these ions are 1414.8, 943.7, and 708.2 respectively consistent with the calculated values. (B) The purified peptide was analyzed on a Gemini-NX C18 column by high performance liquid chromatography (HPLC). It can be seen from the results of HPLC that there is almost only one new predominant product peak, which can prove that the peptide purity is high. The main peak (retention time 11.39 min) corresponds to the target peptide KIMNEBP, and the calculated purity based on peak area is 95.74%.


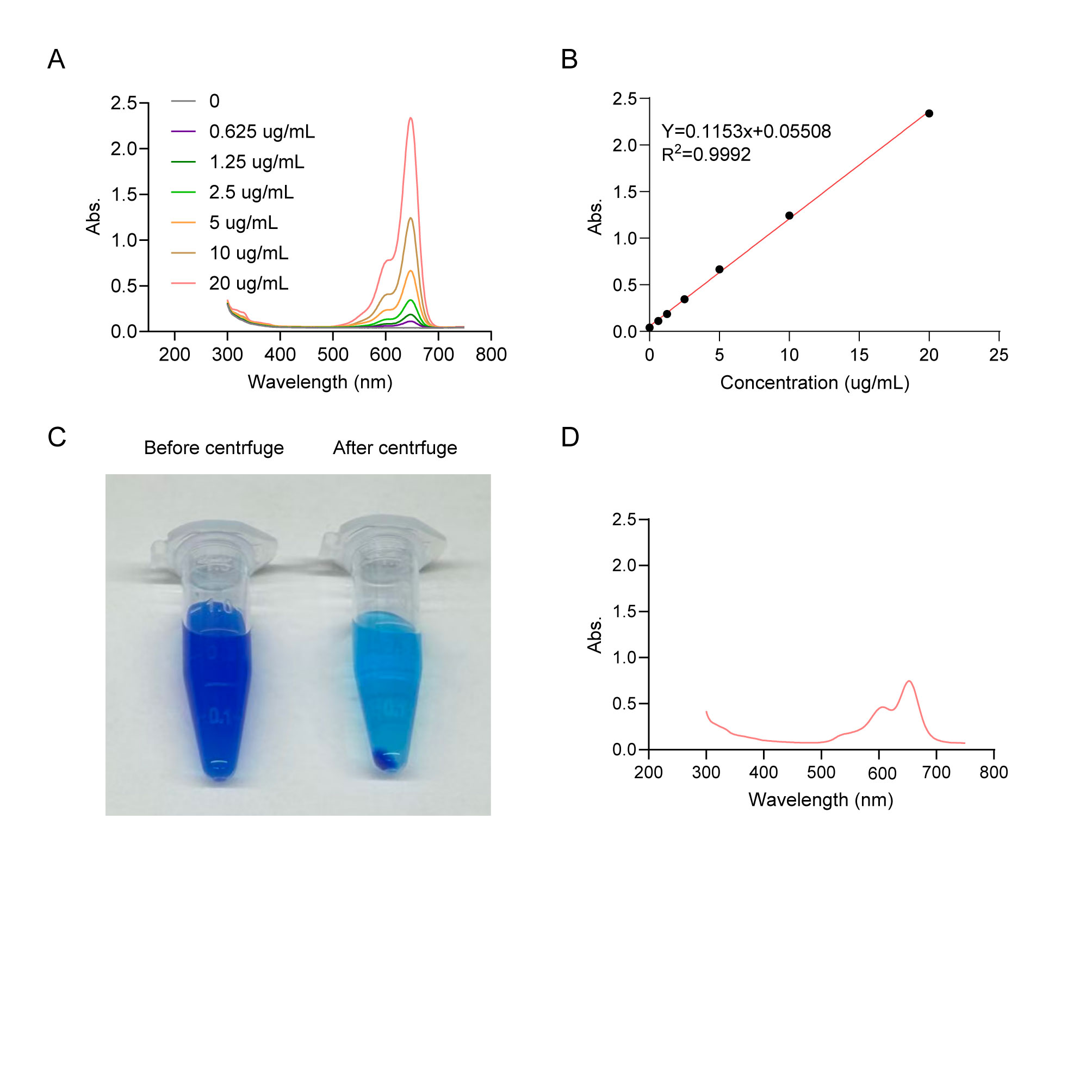


**Figure S3.** UV–visible characterization of Cy5 as a fluorescent surrogate for NMN encapsulation and release from NKN-LNPs. (A) UV-visible absorption spectra of Cy5 at different concentration. (B)The standard curves of UV–visible absorption values of Cy5.The equation of Cy5’s standard curve is Y = 0.1153X+0.05508. The R2 = 0.9992, where X is the concentrations of Cy5, and Y is the absorbance. The equation of drug loading is: $=\frac{100-\frac{Y-0.05508}{0.1153}}{1000}$; where the mass of total Cy5 is 100 μg, the mass of total LNP is 900 μg, and Y is the absorbance of the Cy5 in the supernatant. The equation drug encapsulation yield is: $=$ $\left( 100-\frac{Y-0.05508}{0.1153} \right)*100\%$. (C) Pictures of nanoparticles before and after centrifugation. (D) UV-visible absorption spectra of Cy5 in the supernatant.


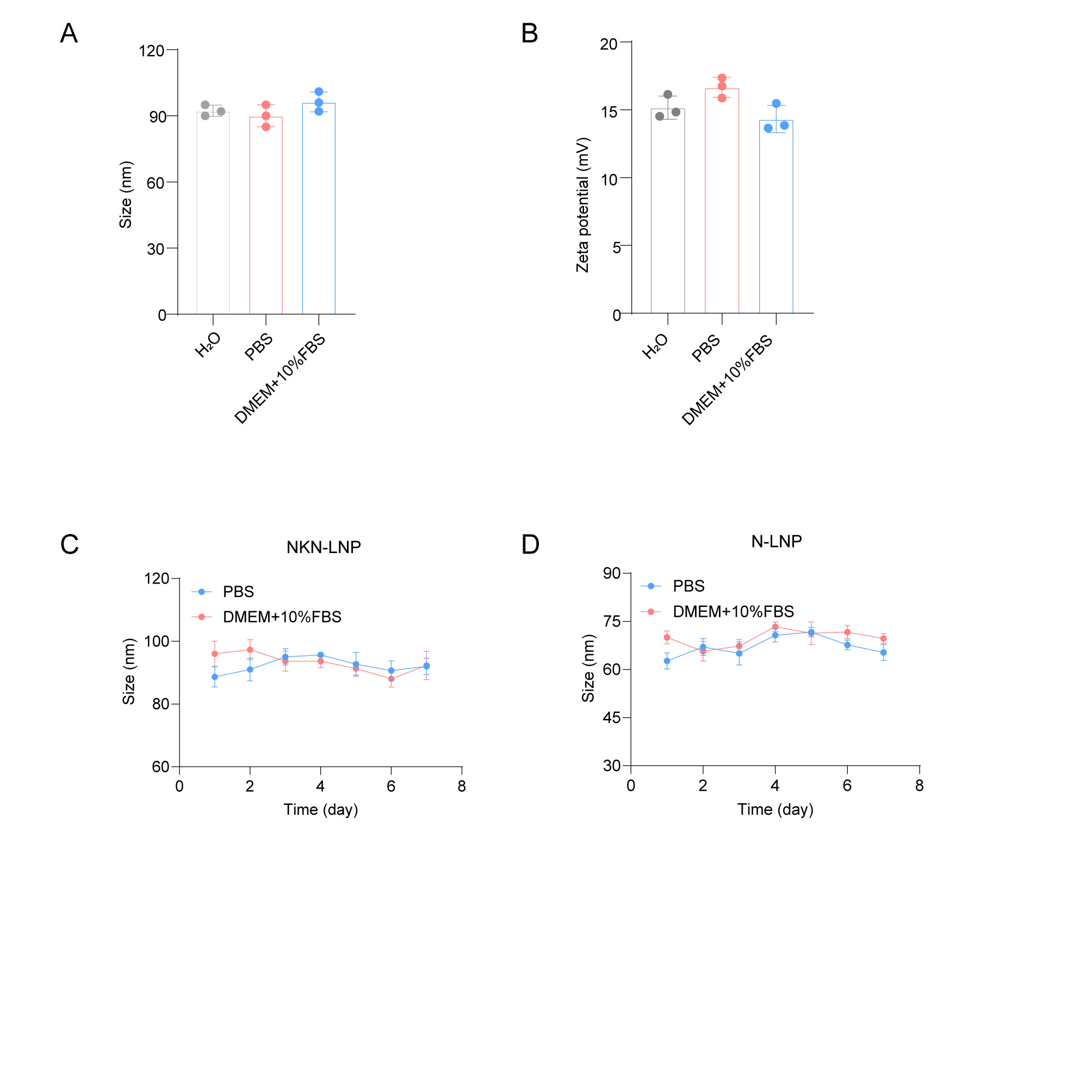


Figure S4. Colloidal stability and physicochemical characterization of NKN-LNPs in physiologically relevant media. (A) Size distribution of NKN-LNPs in H₂O, PBS, and DMEM supplemented with 10% FBS after 12 h incubation. (B) Zeta potential of NKN-LNPs in H₂O, PBS, and DMEM supplemented with 10% FBS after 12 h incubation. (C) Stability of N-LNPs in PBS and DMEM solution during 7 days. (D) Stability of NKN-LNPs in PBS and DMEM solution during 7 days.


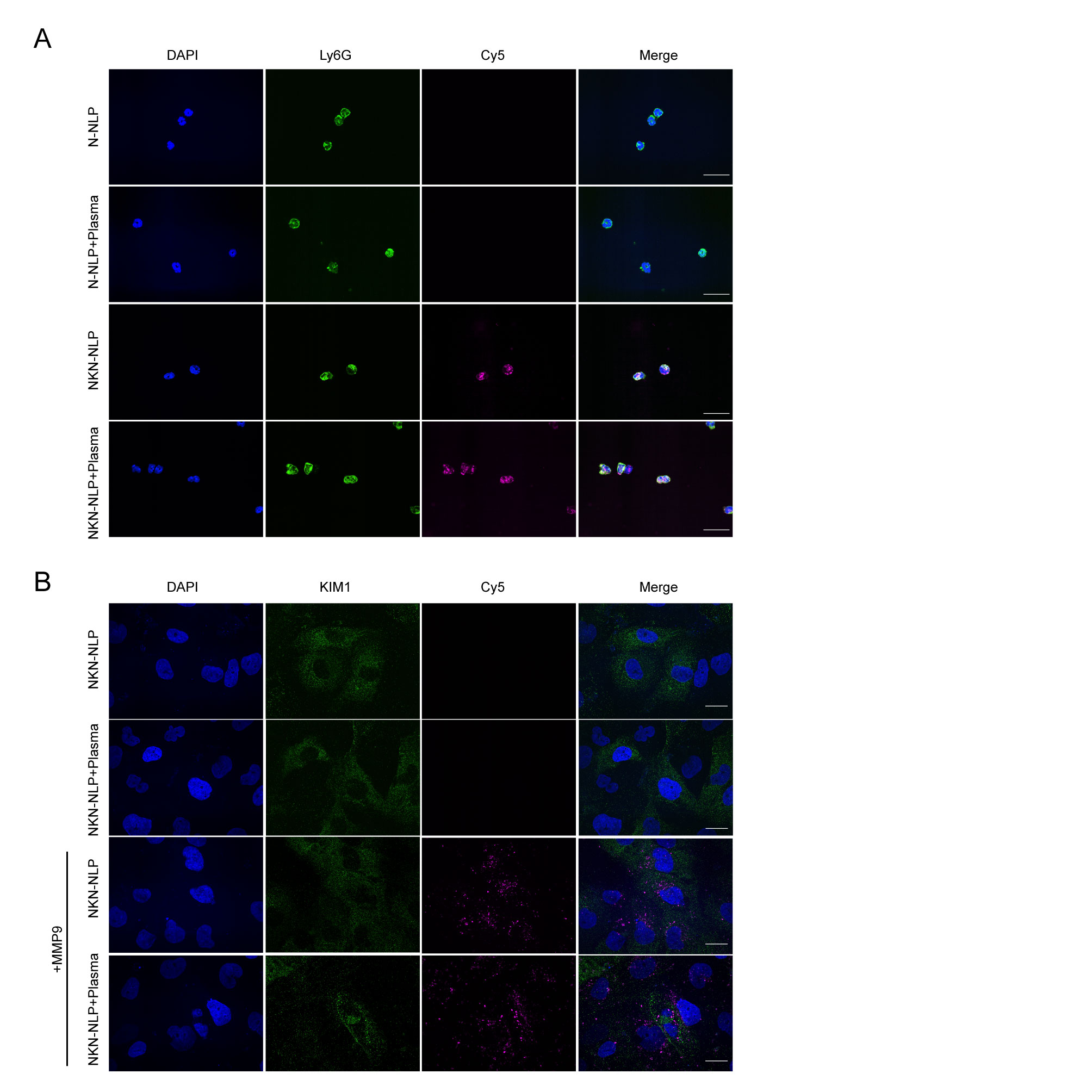


Figure S5. Plasma stability–dependent preservation of neutrophil targeting and MMP-9–responsive KIM1 targeting by NKN-LNPs. (A) Confocal fluorescence images showing neutrophil targeting of liposomes after plasma incubation. Mouse plasma collected from healthy mice was incubated with N-LNPs or NKN-LNPs for 4 h prior to cellular studies. Cells were stained with DAPI (blue, nuclei) and Ly6G (green, neutrophils), while Cy5 (magenta) was used as a fluorescent probe to track liposome uptake. Scale bar:10 μm. (B) Confocal fluorescence images showing MMP-2/9–responsive KIM1 targeting after plasma incubation. Following plasma exposure, liposomes were applied to cells in the presence of MMP-2/9 to evaluate enzyme-responsive targeting. Non-targeted N-LNPs served as controls to distinguish peptide-mediated targeting from nonspecific uptake. Scale bar: 10 μm.


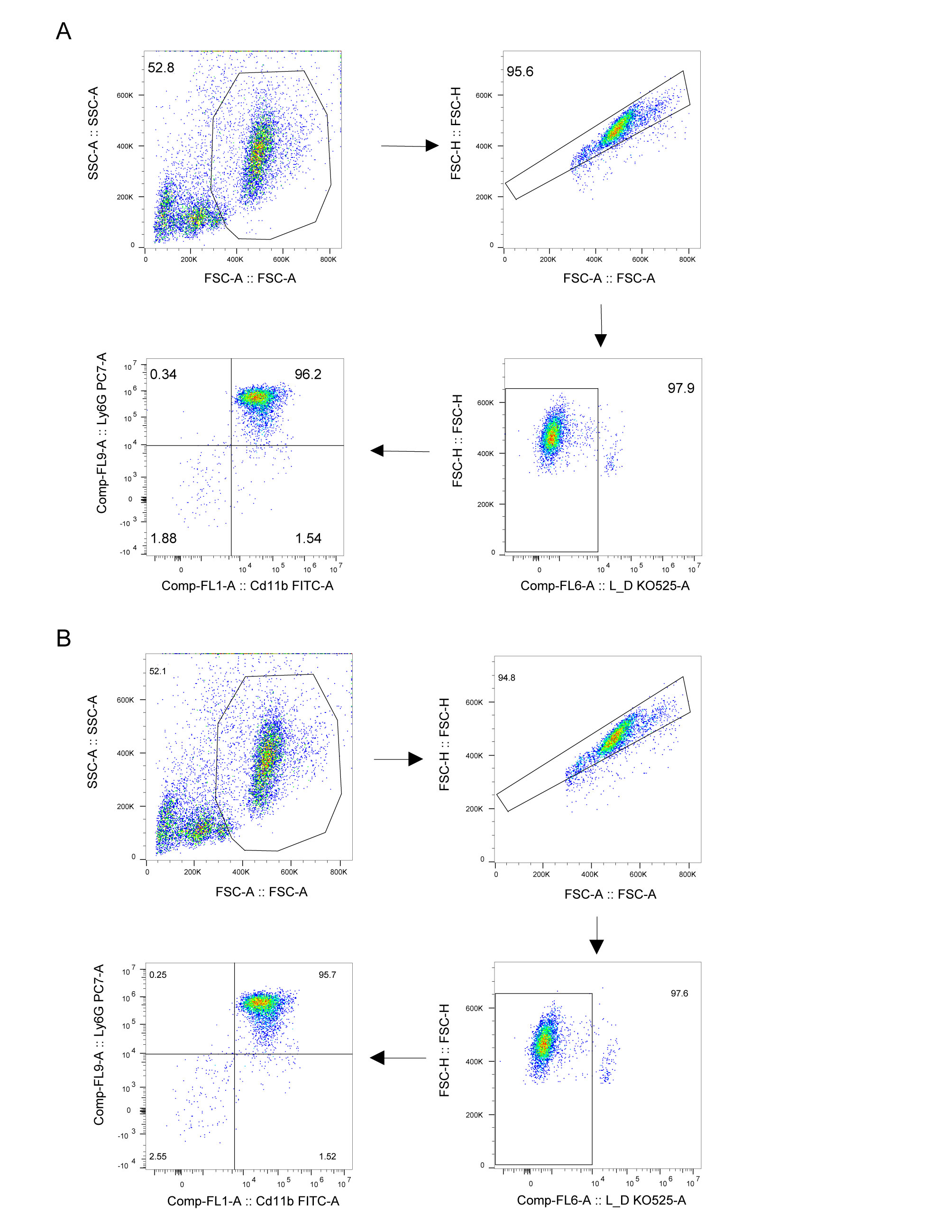


**Figure S6.** Flow cytometric analysis of neutrophil identification, purity, and NKN-LNP uptake. (A) Purity assessment of bone marrow-derived neutrophils. Representative flow cytometry plot showing the high purity of isolated neutrophils, as defined by CD11b and Ly6G co-expression. (B) Uptake of NKN-LNPs by neutrophils in vivo. Quantitative analysis of Cy5⁺ signal within peripheral blood neutrophils from UIRIx mice treated with PBS (Control), N-LNP, or Cy5-labeled NKN-LNP.


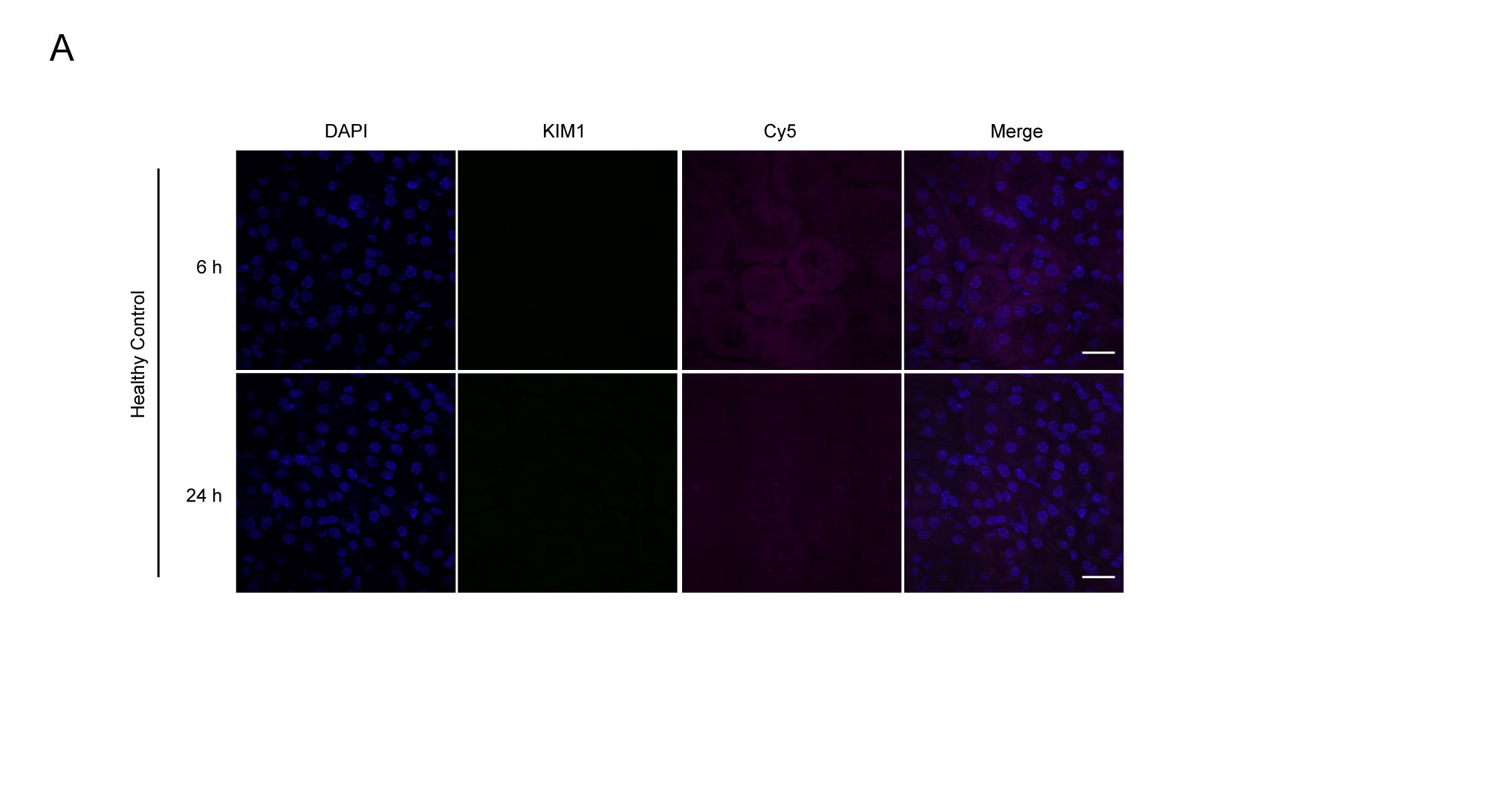


**Figure S7A.** Representative immunofluorescence images of healthy kidney tissues at 6 h and 24 h after administration of Cy5-labeled NKN-LNP. Nuclei were stained with DAPI (blue), KIM1 was detected by immunofluorescence (green), and NKN-LNP was visualized by Cy5 fluorescence (magenta). Scale bar: 10 μm.


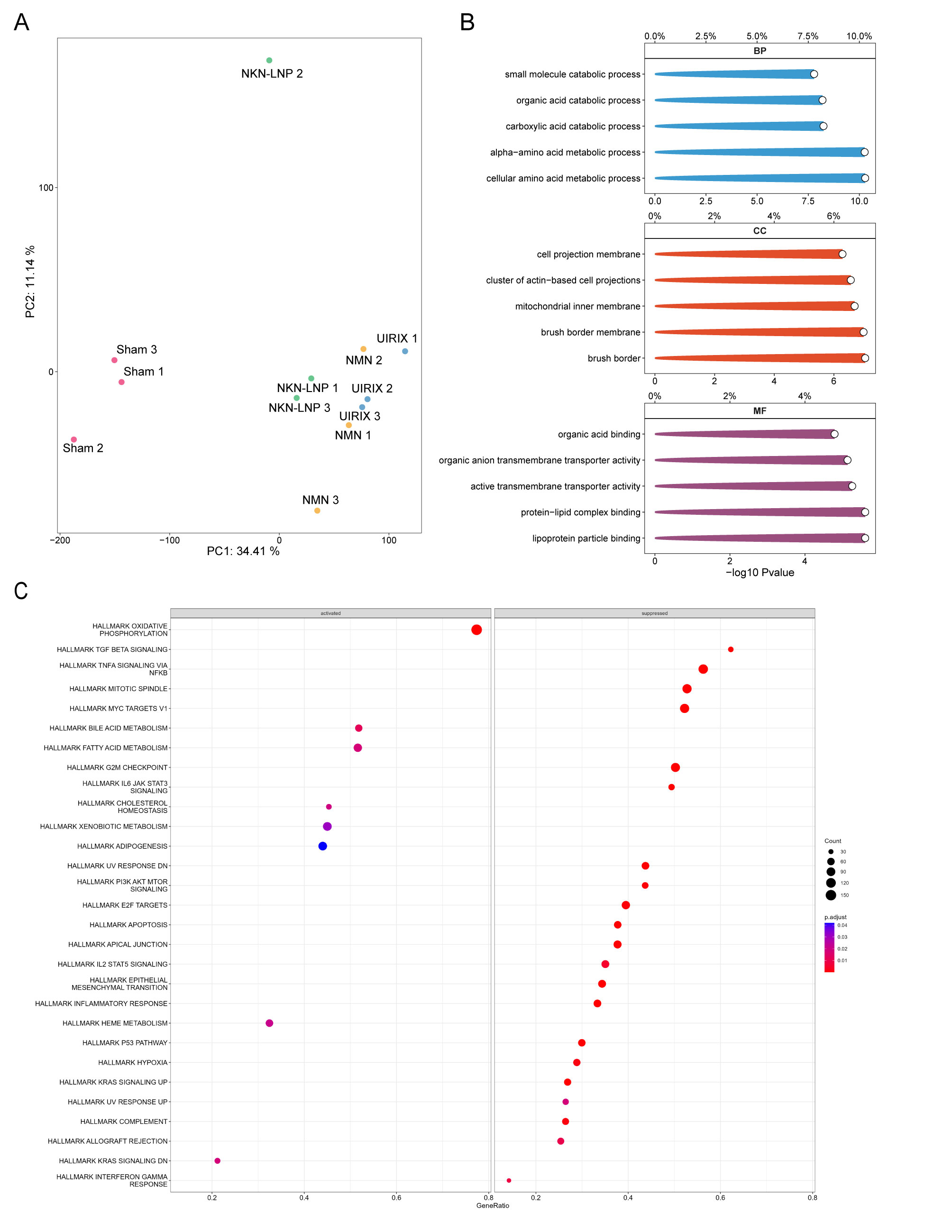


**Figure S8.** Integrated bioinformatics analysis identifies distinct profiles and functional pathways. (A) Principal Component Analysis (PCA) demonstrating the clear separation of transcriptomes among groups, indicating distinct global gene expression patterns.

(B) Results of Gene Ontology (GO) enrichment analysis, highlighting the most significantly overrepresented biological processes, molecular functions, and cellular components. (C) Gene Set Enrichment Analysis (GSEA) results for selected gene sets, confirming the coordinated upregulation or downregulation of key defined pathways.


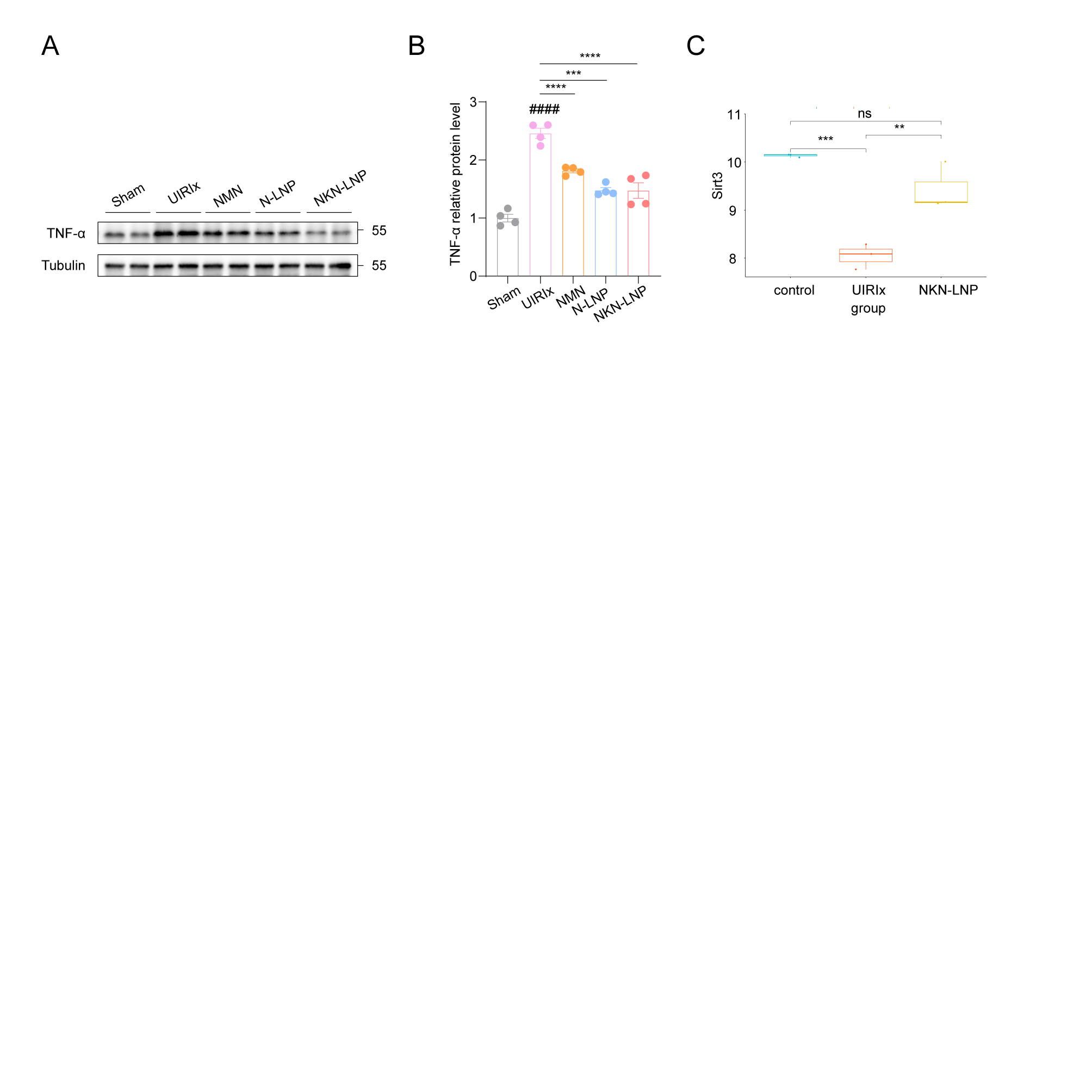
**Figure S9.** NKN-LNP modulates TNF-α protein and SIRT3 mRNA expression in renal ischemia-reperfusion injury (A) Representative Western blot of TNF-α protein expression. (B) Quantification analysis showing suppression of TNF-α protein levels in NKN-LNP group. Data are presented as mean ± SEM; n = 4 per group. Statistics: one-way ANOVA with Tukey's test. Significance: ** p < 0.01, *** p < 0.001 vs. UIRIx; #### p < 0.0001 UIRIx vs. Sham. (C) Transcriptional downregulation of Sirt3 in UIRIx group rescued by NKN-LNP Data are presented as mean ± SEM; n = 3 per group. Statistics: one-way ANOVA with Tukey's test. Significance: * p < 0.05, ** p <0.01, *** p < 0.001, ns, not significant.


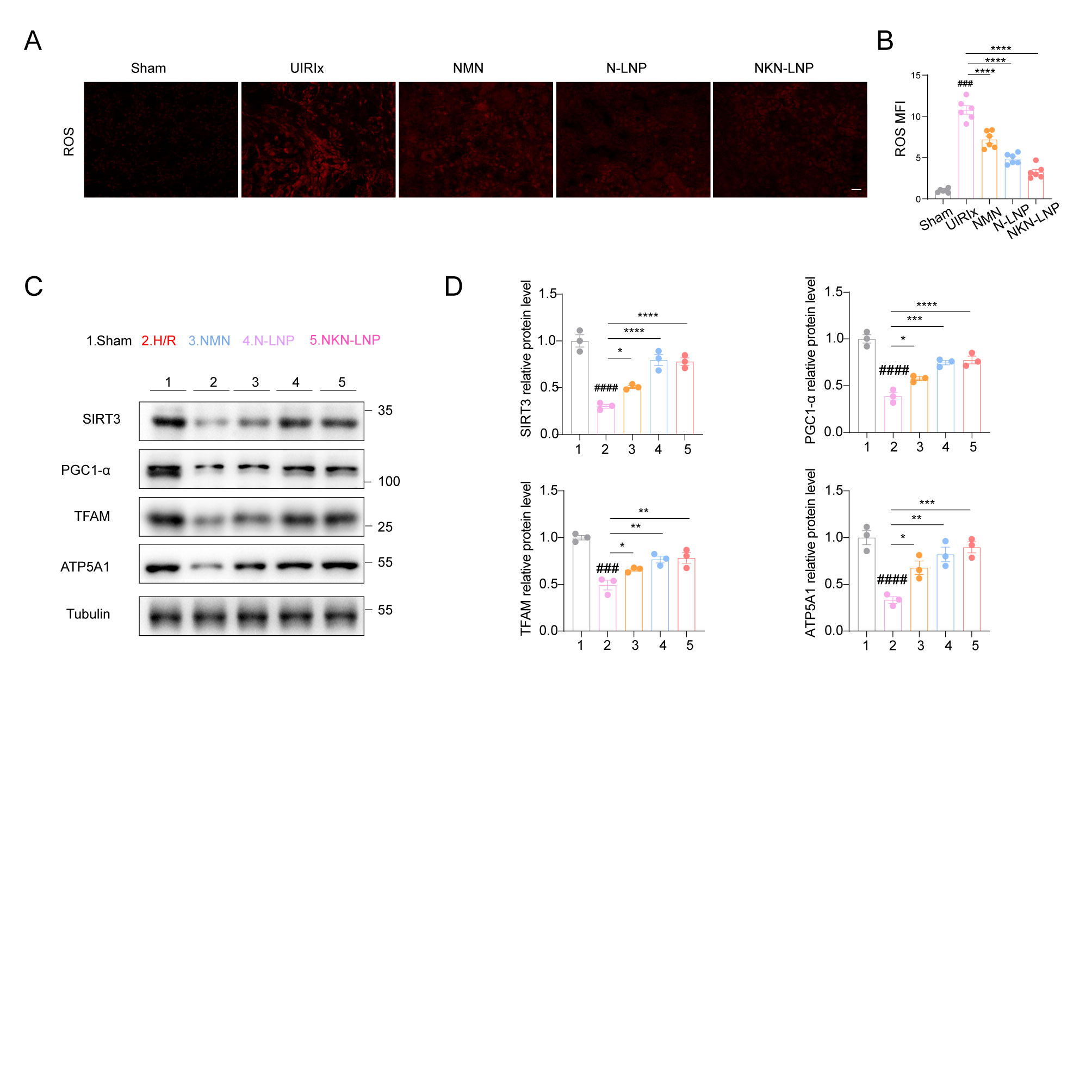


**Figure S10.** Analysis of renal ROS and expression of key mitochondrial proteins (PGC-1α, TFAM, SIRT3, ATP5A1) in H/R-treated HK-2 cells. (A) Representative images of ROS staining in kidney tissues across the different treatment groups (scale bar: 20 μm). (B) Quantification of ROS fluorescence intensity (n = 6 per group). (C) Representative Western blot images, and (D) corresponding quantification of PGC-1α, TFAM, ATP5A1, SIRT3, and Tubulin protein levels in HK-2 H/R model (n = 3 per group). In (B), (D), Data are presented as mean ± SEM. Statistical significance was determined using one-way ANOVA followed by Tukey’s post hoc test. * p < 0.05, ** p < 0.01, *** p < 0.001, **** p < 0.0001 versus UIRIx (H/R). #### p < 0.0001 UIRIx (H/R) versus Sham.


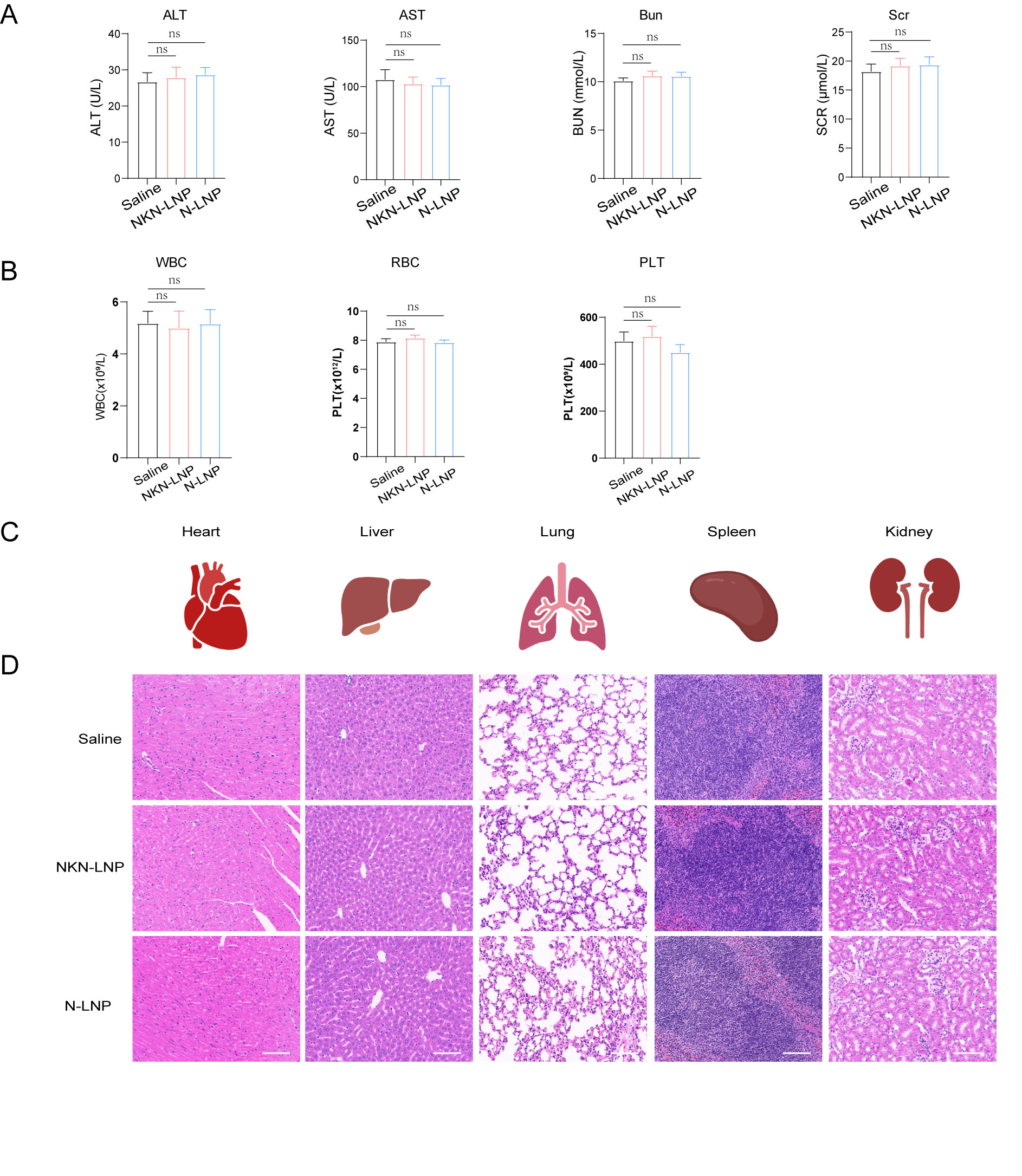


**Figure S11.** Biosafety evaluation of N-LNP and NKN-LNP nanocarriers. (A) Blood levels of ALT, AST, BUN, and SCr in mice after intravenous administration of NKN-LNP or N-LNP (n = 3 per group). Statistics: one-way ANOVA with Tukey's test. Data are presented as mean ± SEM. ns, not significant. (B) Routine blood analysis of mice after a single tail-vein injection of saline, non-targeted N-LNPs, or NKN-LNPs. White blood cell count (WBC), red blood cell count (RBC), and platelet count (PLT) showed no significant differences among the three groups. Data are presented as mean ± SEM (n = 3). One-way ANOVA was used for statistical analysis. (C) Schematic illustration of the major organs (heart, liver, lung, spleen, and kidney). Created in BioRender. Chu, F. (2025) <https://BioRender.com/ckd70ec>. (D) Representative H&E staining of histological sections from major organs was performed 14 days following intravenous administration of Saline, NKN-LNP, or N-LNP in healthy mice (n = 3 per group). Scale bar:100 μm.
