# Supplementary figures and images for: Neutrophil‐Driven Cascade‐Targeted Nanocarriers Restore Mitochondrial Homeostasis to Ameliorate Renal Ischemia–Reperfusion Injury
Source: Adv Sci (Weinh). 2026 Mar 30;13(30):e20940. doi: 10.1002/advs.202520940 (PMC13248797; doi:10.1002/advs.202520940)

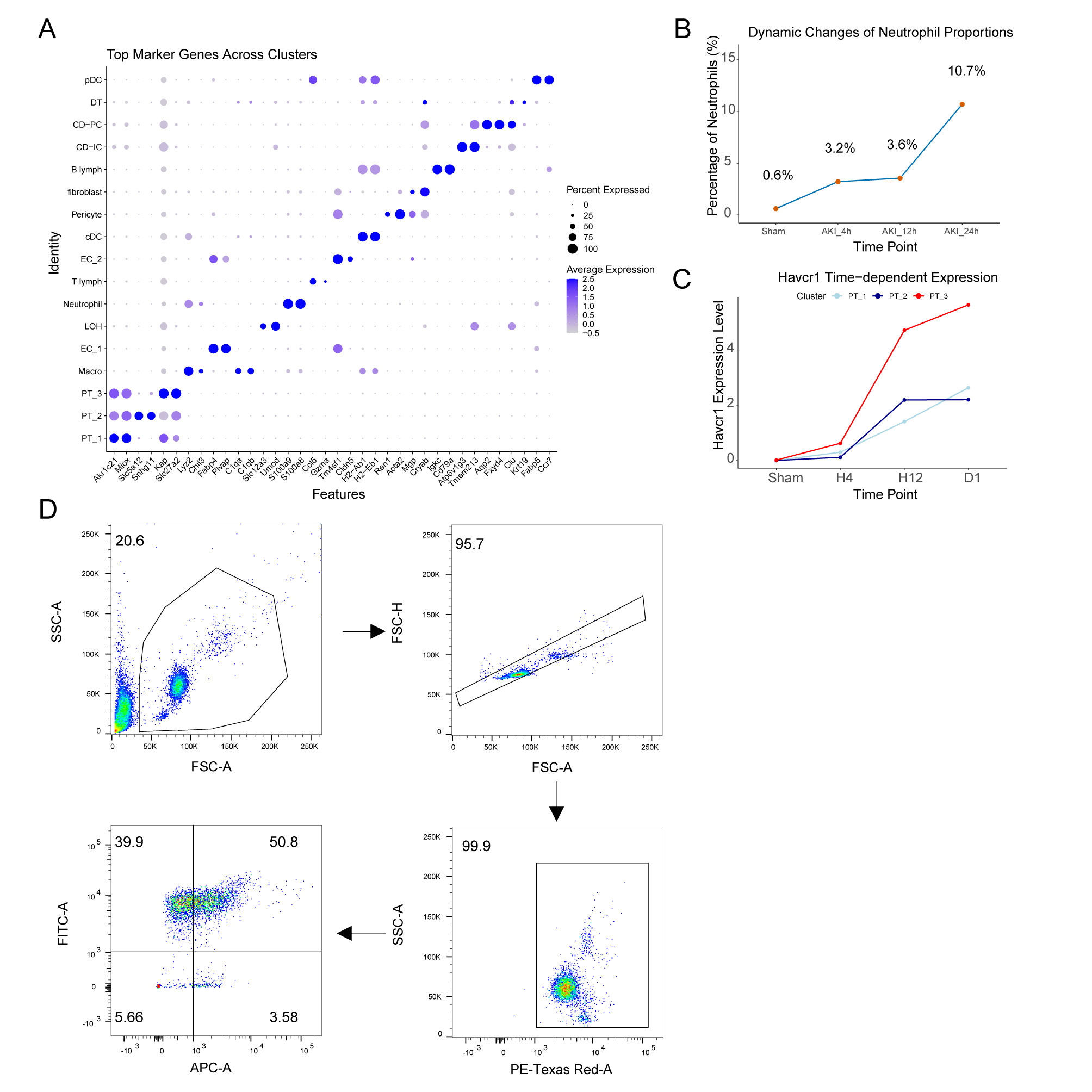

Supplement: Supplementary file 2 — Supporting File 2: advs74824‐sup‐0002‐FigureS1‐S11.zip. [file ADVS-13-e20940-s002.zip › FIGURES1.jpg]

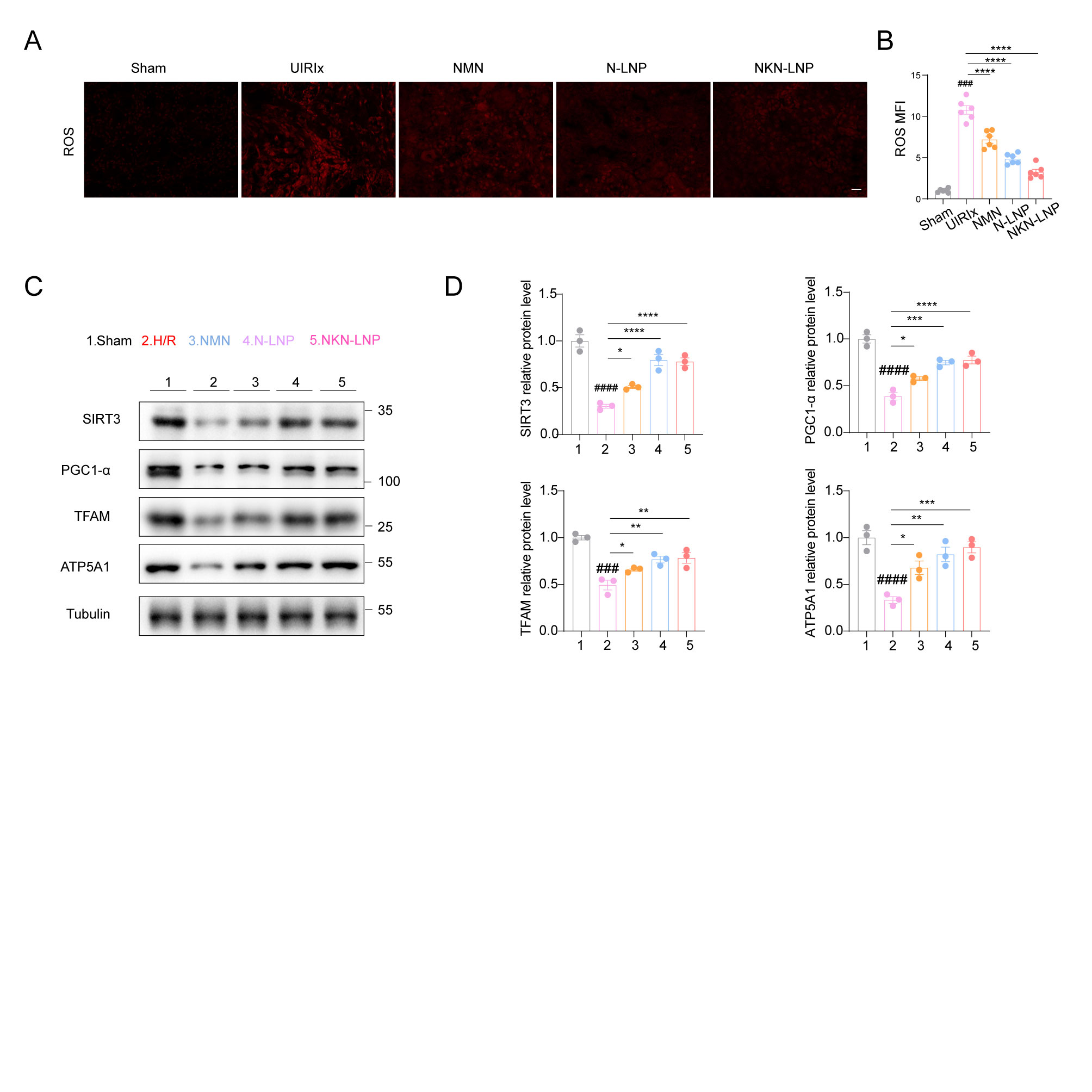

Supplement: Supplementary file 2 — Supporting File 2: advs74824‐sup‐0002‐FigureS1‐S11.zip. [file ADVS-13-e20940-s002.zip › FigureS10.jpg]

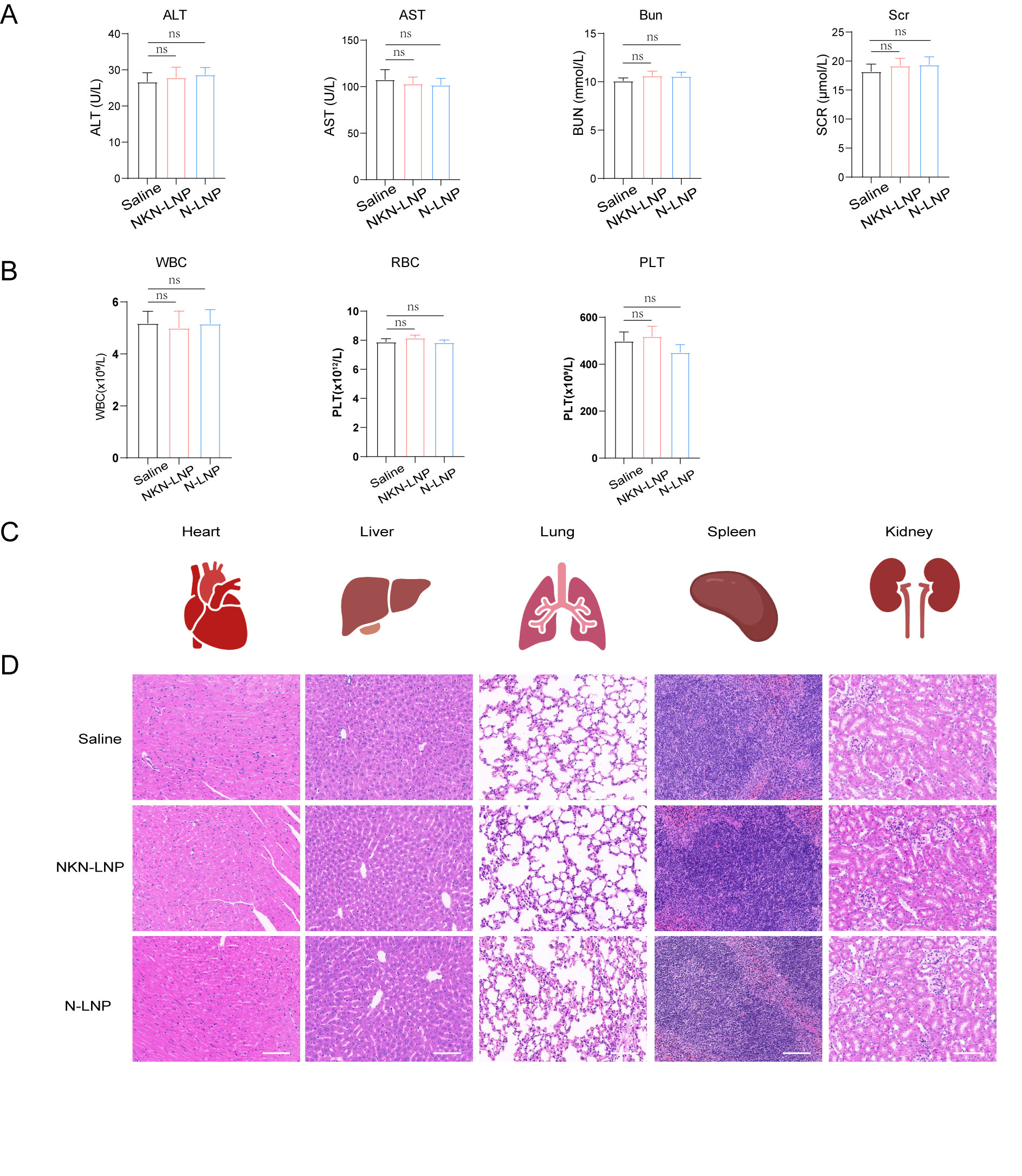

Supplement: Supplementary file 2 — Supporting File 2: advs74824‐sup‐0002‐FigureS1‐S11.zip. [file ADVS-13-e20940-s002.zip › FigureS11.jpg]

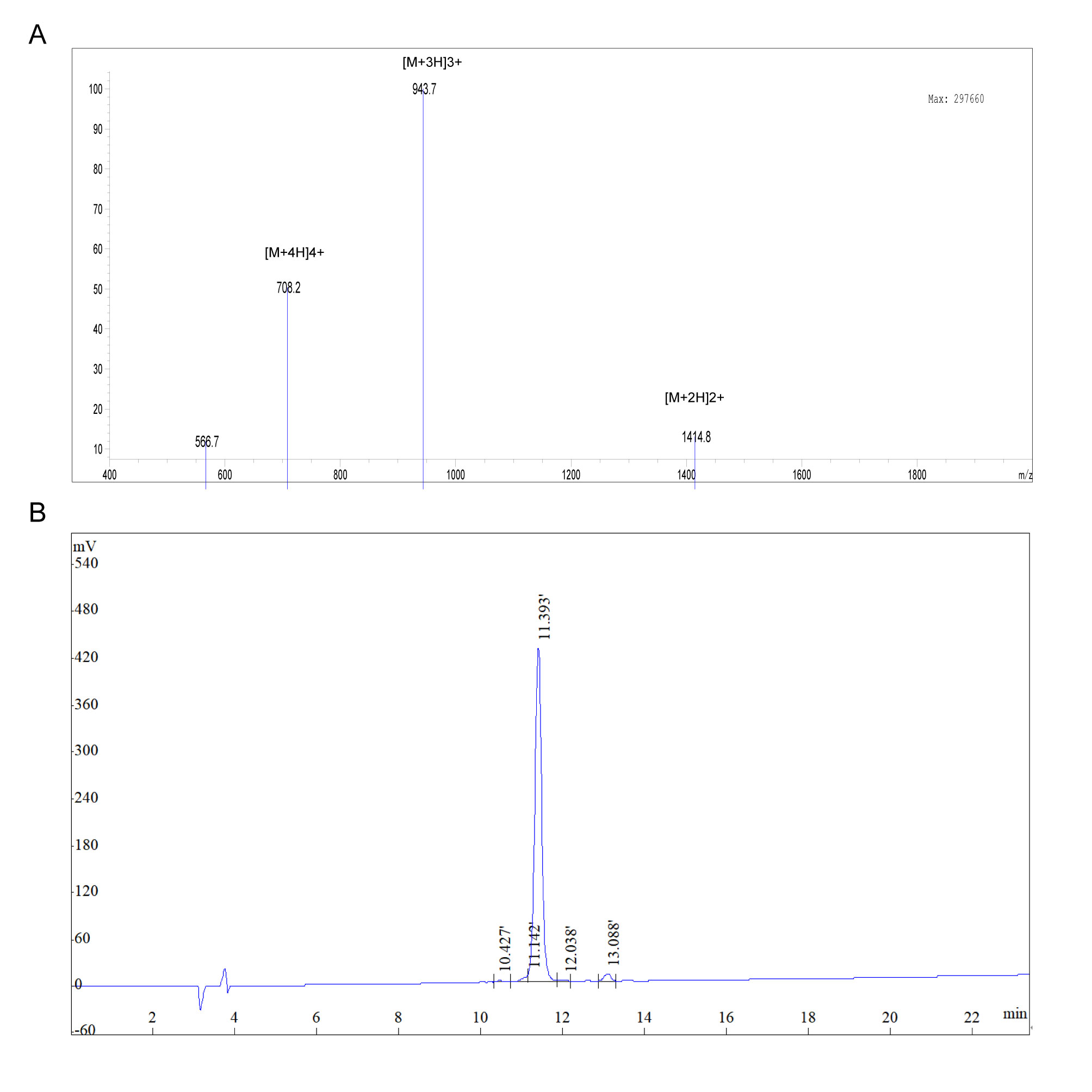

Supplement: Supplementary file 2 — Supporting File 2: advs74824‐sup‐0002‐FigureS1‐S11.zip. [file ADVS-13-e20940-s002.zip › FigureS2.jpg]

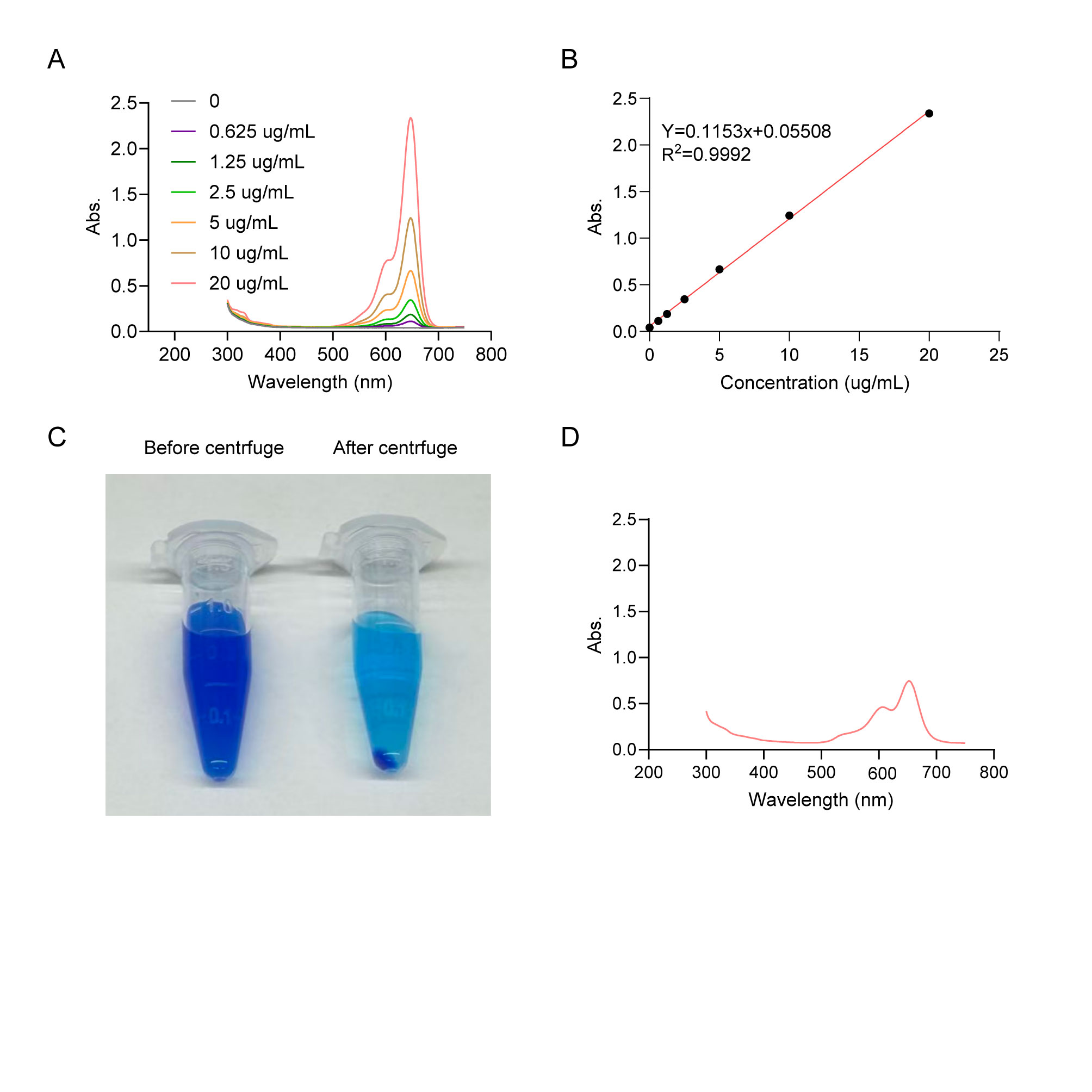

Supplement: Supplementary file 2 — Supporting File 2: advs74824‐sup‐0002‐FigureS1‐S11.zip. [file ADVS-13-e20940-s002.zip › FigureS3.jpg]

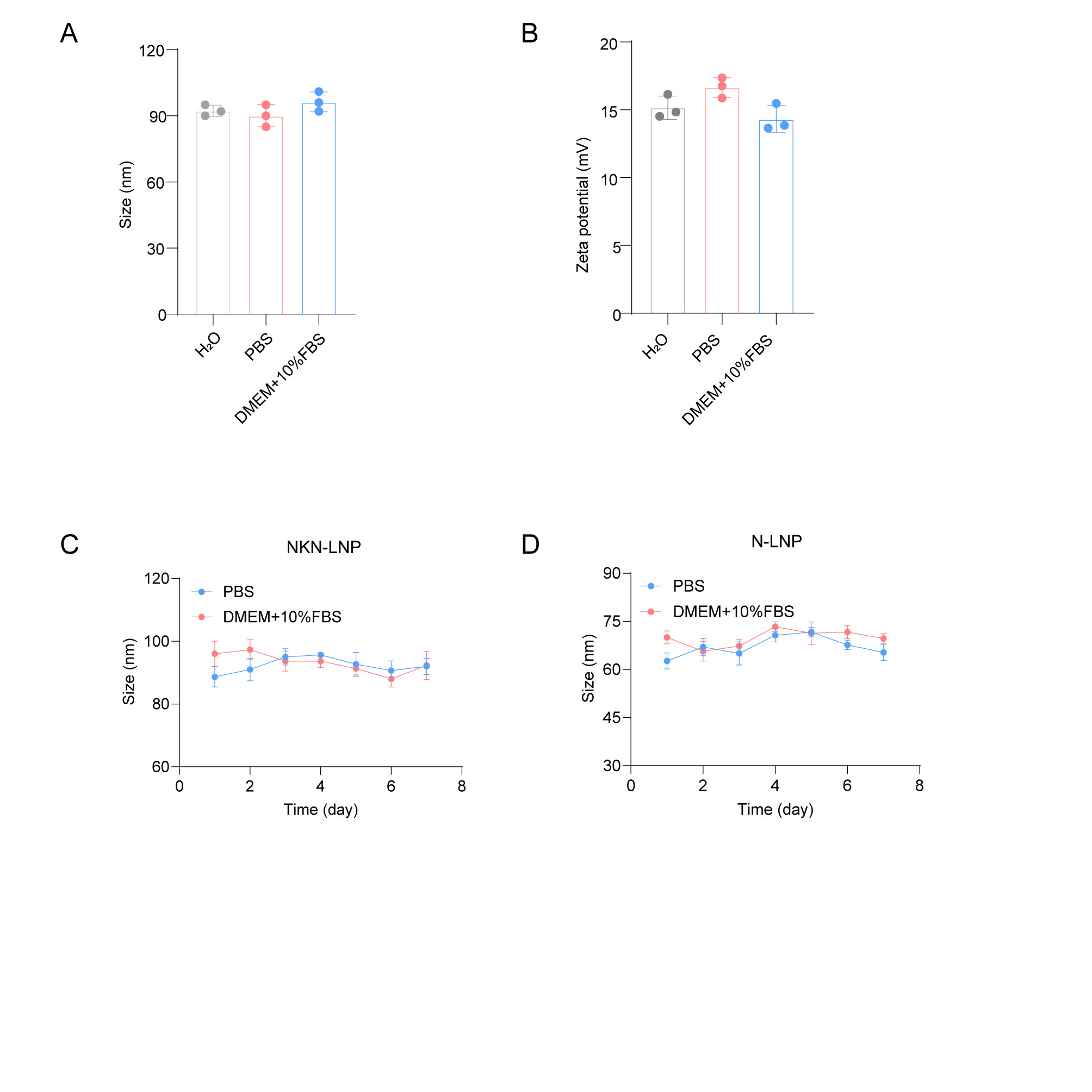

Supplement: Supplementary file 2 — Supporting File 2: advs74824‐sup‐0002‐FigureS1‐S11.zip. [file ADVS-13-e20940-s002.zip › FigureS4.jpg]

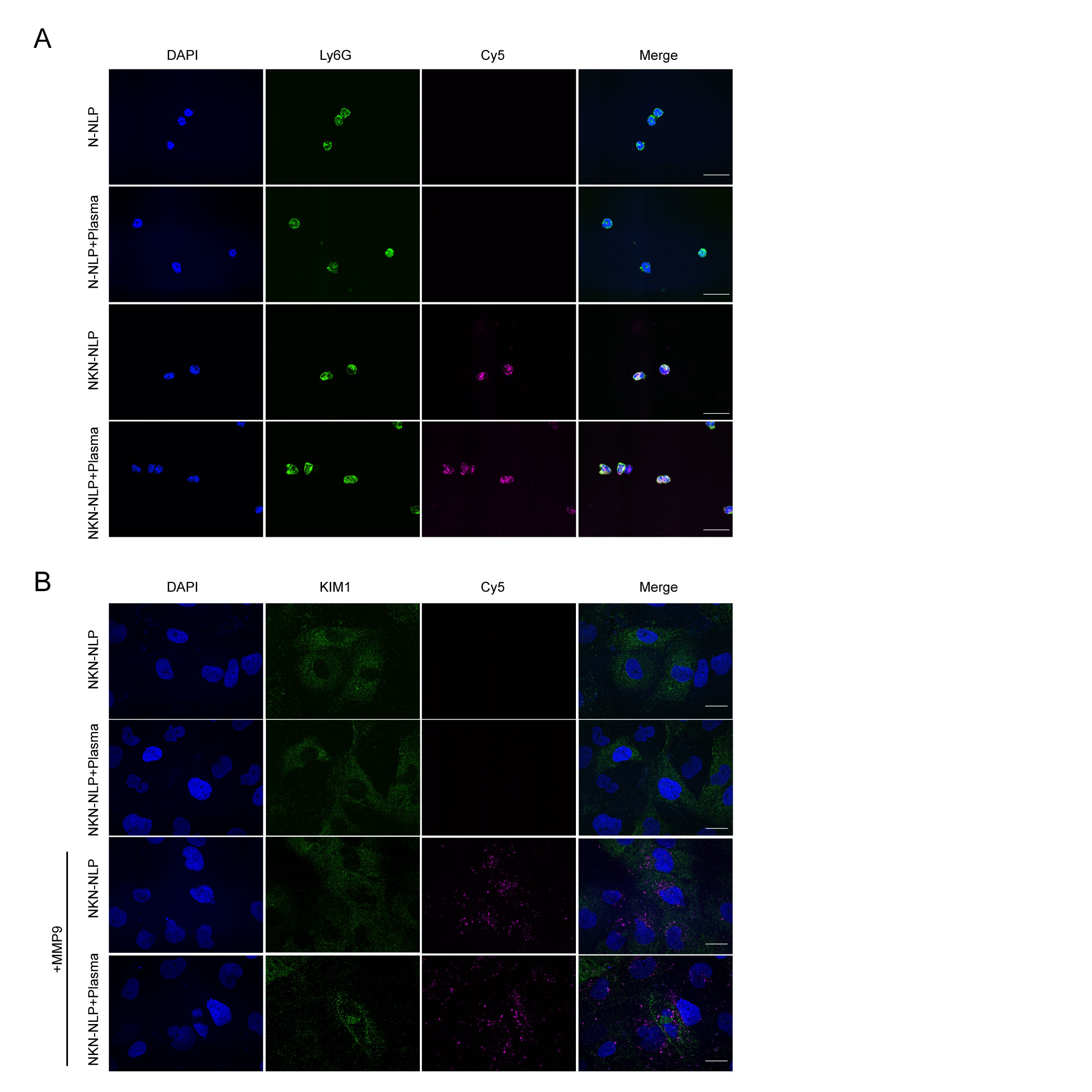

Supplement: Supplementary file 2 — Supporting File 2: advs74824‐sup‐0002‐FigureS1‐S11.zip. [file ADVS-13-e20940-s002.zip › FigureS5.jpg]

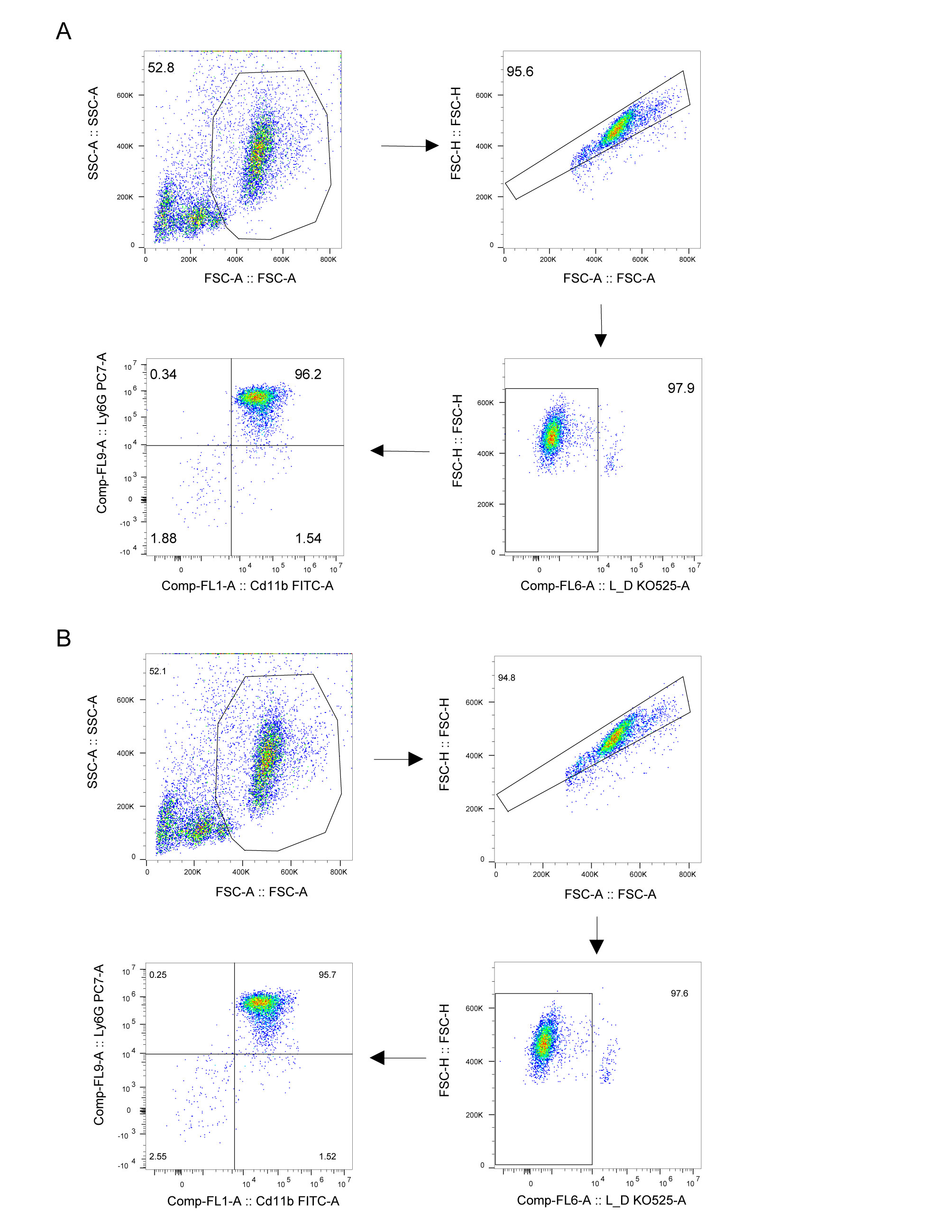

Supplement: Supplementary file 2 — Supporting File 2: advs74824‐sup‐0002‐FigureS1‐S11.zip. [file ADVS-13-e20940-s002.zip › FigureS6.jpg]

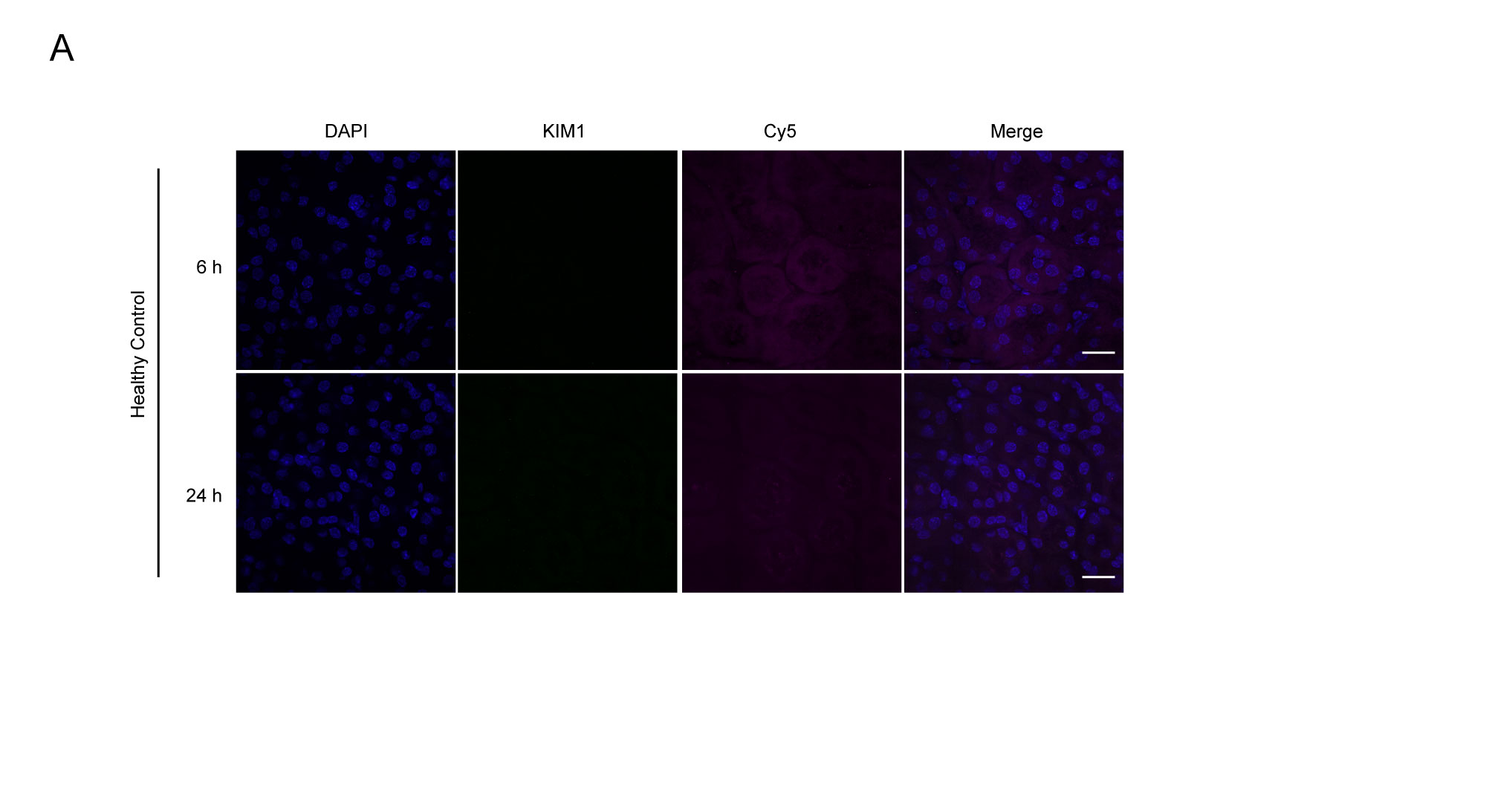

Supplement: Supplementary file 2 — Supporting File 2: advs74824‐sup‐0002‐FigureS1‐S11.zip. [file ADVS-13-e20940-s002.zip › FigureS7.jpg]

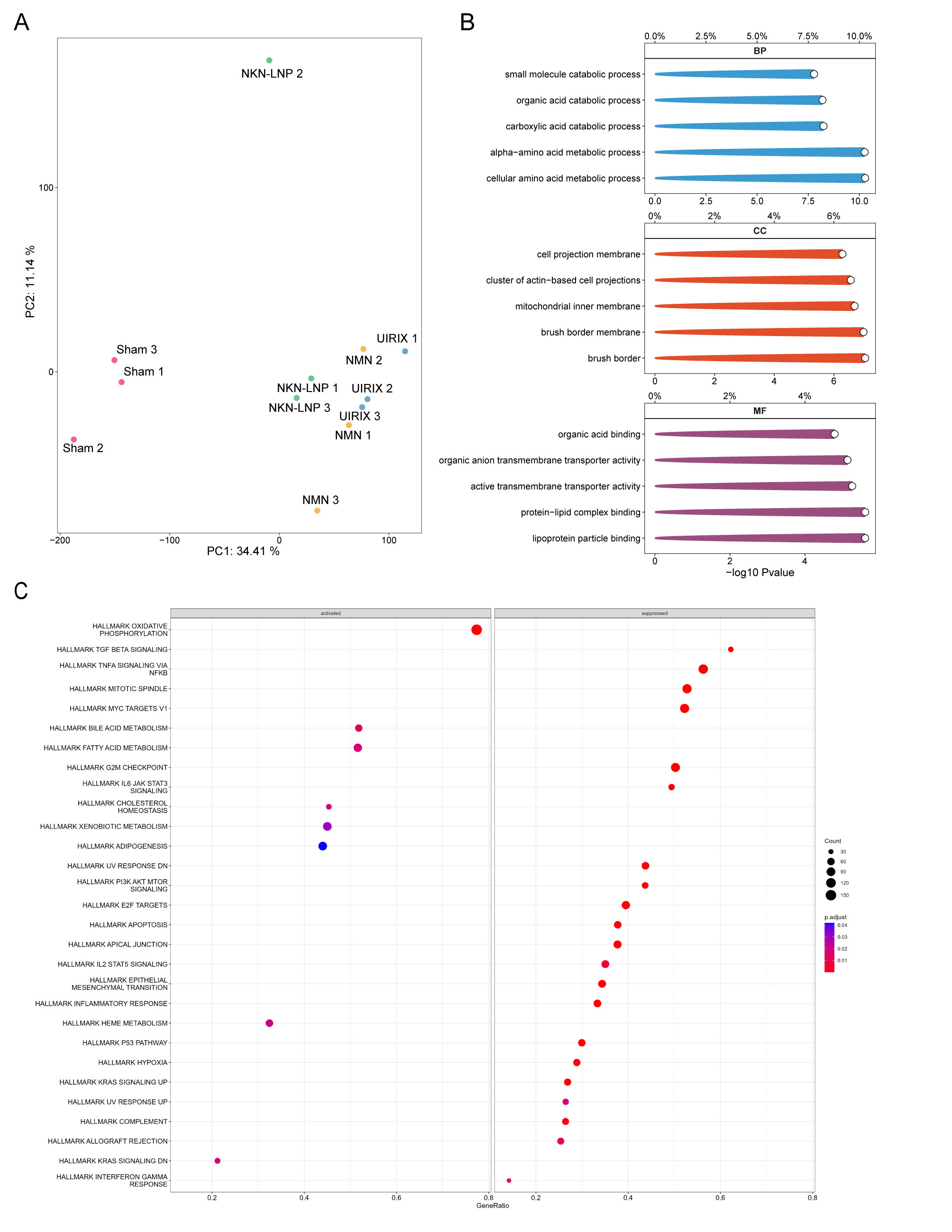

Supplement: Supplementary file 2 — Supporting File 2: advs74824‐sup‐0002‐FigureS1‐S11.zip. [file ADVS-13-e20940-s002.zip › FigureS8.jpg]

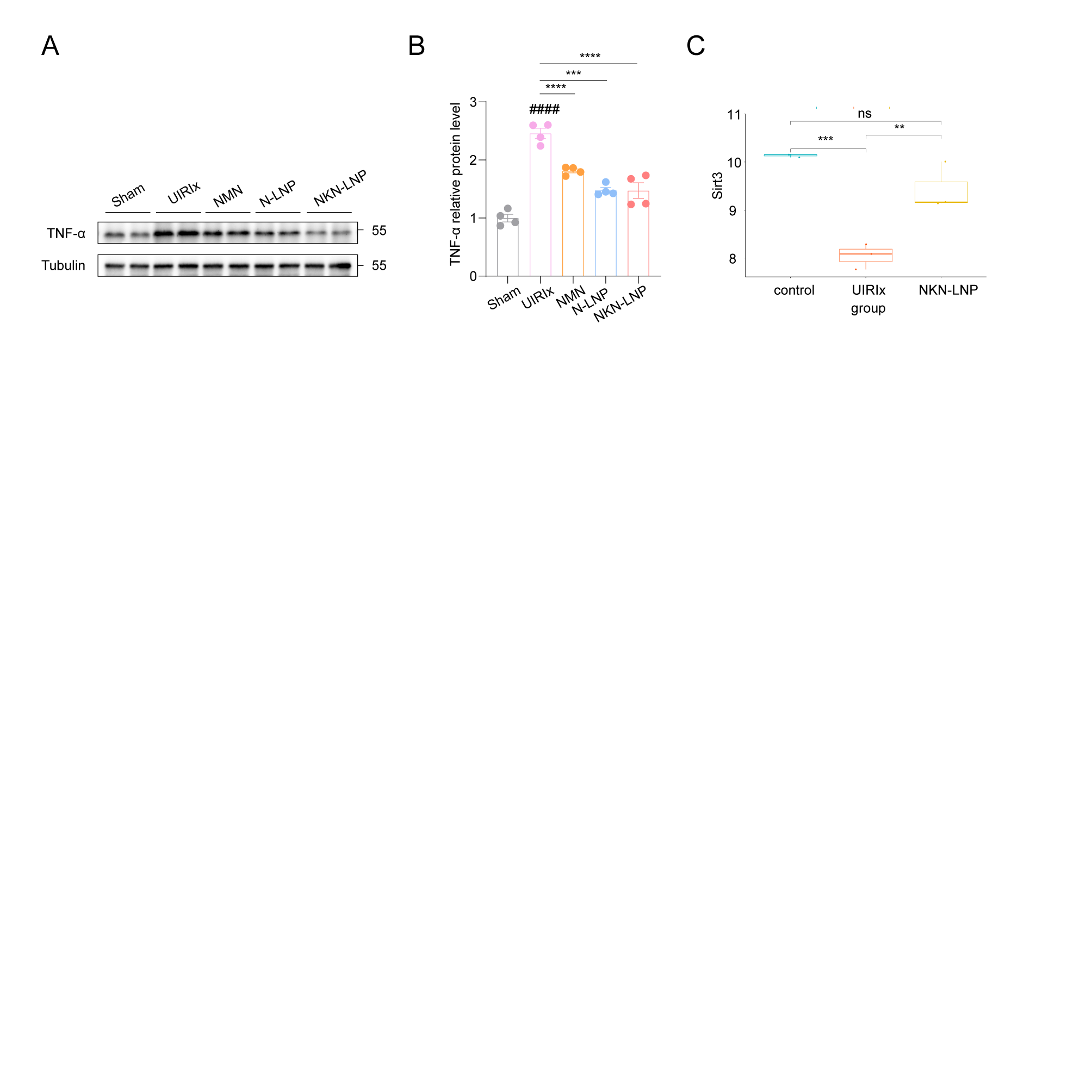

Supplement: Supplementary file 2 — Supporting File 2: advs74824‐sup‐0002‐FigureS1‐S11.zip. [file ADVS-13-e20940-s002.zip › FigureS9.tif]
