# Supplementary material for: Neutrophil‐Driven Cascade‐Targeted Nanocarriers Restore Mitochondrial Homeostasis to Ameliorate Renal Ischemia–Reperfusion Injury
Source: Adv Sci (Weinh). 2026 Mar 30;13(30):e20940. doi: 10.1002/advs.202520940 (PMC13248797; doi:10.1002/advs.202520940)
Supplement: Supplementary file 3 — Supporting File 3: advs74824‐sup‐0003‐TableS1‐S2.docx. [file ADVS-13-e20940-s001.docx]

**TABLE S1**

| Antibodies | | |
| --- | --- | --- |
| Rabbit polyclonal anti-KIM1 | Proteintech | 30948-1-AP |
| Rabbit monoclonal anti- BCL-2 | Proteintech | 26593-1-AP |
| Rabbit monoclonal anti-BAX | Proteintech | 26765-1-AP |
| Mouse monoclonal anti-GAPDH | Proteintech | 60004-1-lg |
| Rabbit monoclonal anti- Tubulin | Proteintech | 66031-1-Ig |
| Rabbit monoclonal anti- Sirt3 | HUABIO | HA722251 |
| Mouse monoclonal anti-PGC1-α | Proteintech | 66369-1-Ig |
| Mouse monoclonal anti-Atp5a1 | Proteintech | 66037-1-Ig |
| Rabbit monoclonal anti-TFAM | Proteintech | 68557-5-Ig |
| Rabbit polyclonal anti- Myeloperoxidase | Abcam | EPR20257 |
| Rabbit polyclonal anti-TNFα | Proteintech | 17590-1-AP |
| Rabbit polyclonal anti-Fibronectin | Abcam | ab2413 |
| Rabbit polyclonal anti-Vimentin | Proteintech | 10366-1-AP |
| Rabbit polyclonal anti- COL1A1 | CST | 72026 |
| HRP-conjugated Affinipure Goat Anti-Rabbit IgG(H+L) | Proteintech | SA00001-2 |
| HRP-conjugated Affinipure Goat Anti-Mouse IgG(H+L) | Proteintech | SA00001-1 |
| Rat monoclonal anti-Ly6G | CST | 88876 |
| Rat monoclonal anti-CD11b | CST | 41249 |
| Rabbit polyclonal anti-KIM1（IF cell） | Abcam | MAB1750 |
| Rabbit polyclonal anti-NGAL | Proteintech | 30700-1-AP |
| α-SMA | CST | 19245 |

**TABLE S2**

| **Alterations in Peripheral Blood Neutrophil Percentage in Patients Undergoing Partial Nephrectomy** | | |
| --- | --- | --- |
| Patient ID | Before (Neutrophil Percentage %) | After (Neutrophil Percentage %) |
| 1 | 56.4 | 78.6 |
| 2 | 63.6 | 73.8 |
| 3 | 53.8 | 79.6 |
| 4 | 72.4 | 85.2 |
| 5 | 60.6 | 92.1 |
| 6 | 61.3 | 86.0 |
| 7 | 64.3 | 92.3 |
| 8 | 50.0 | 80.5 |
| 9 | 65.2 | 75.1 |
| 10 | 56.8 | 73.2 |
| 11 | 59.1 | 79.6 |
| 12 | 67.7 | 85.2 |
| 13 | 60.0 | 86.0 |
| 14 | 71.7 | 87.7 |
| 15 | 54.1 | 77.4 |
| 16 | 59.9 | 81.6 |
| 17 | 58.4 | 76.6 |
| 18 | 58.5 | 81.3 |
| 19 | 57.6 | 85.3 |
| 20 | 61.2 | 91.1 |
| 21 | 67.2 | 86.5 |
| 22 | 51.0 | 77.8 |
| 23 | 57.9 | 85.8 |
| 24 | 62.7 | 89.1 |
| 25 | 60.3 | 92.3 |
| 26 | 68.0 | 84.6 |
| 27 | 70.0 | 81.0 |
| 28 | 61.9 | 83.7 |
| 29 | 59.8 | 85.1 |
